# Installation of click-type functional groups enable the creation of an additive manufactured construct for the osteochondral interface

**DOI:** 10.1088/1758-5090/aca3d4

**Published:** 2022-12-15

**Authors:** Ivo A O Beeren, Pieter J Dijkstra, Ana Filipa H Lourenço, Ravi Sinha, David B Gomes, Hong Liu, Nicole Bouvy, Matthew B Baker, Sandra Camarero-Espinosa, Lorenzo Moroni

**Affiliations:** 1 Department of Complex Tissue Regeneration, MERLN Institute for Technology-Inspired Regenerative Medicine, Maastricht University, Maastricht, 6229 ER, The Netherlands; 2 Department of General Surgery, Maastricht University Medical Center, 6200 ND Maastricht, The Netherlands; 3 POLYMAT, University of the Basque Country UPV/EHU, Avenida Tolosa 72, Donostia/San Sebastián 20018 Gipuzkoa, Spain; 4 IKERBASQUE, Basque Foundation for Science, Bilbao 48009, Spain

**Keywords:** additive manufacturing, scaffolds, gradients, orthogonal chemistry, osteochondral regeneration, peptides

## Abstract

Melt extrusion-based additive manufacturing (AM) is often used to fabricate scaffolds for osteochondral (OC) regeneration. However, there are two shortcomings associated with this scaffold manufacturing technique for engineering of tissue interfaces: (a) most polymers used in the processing are bioinert, and (b) AM scaffolds often contain discrete (material) gradients accompanied with mechanically weak interfaces. The inability to mimic the gradual transition from cartilage to bone in OC tissue leads to poor scaffold performance and even failure. We hypothesized that introducing peptide gradients on the surface could gradually guide human mesenchymal stromal cell (hMSC) differentiation, from a chondrogenic towards on osteogenic phenotype. To work towards this goal, we initially manufactured poly(*ε*-caprolactone)-azide (PCLA) and PCL-maleimide (PCLM) scaffolds. The surface exposed click-type functional groups, with a surface concentration in the 10^2^ pmol cm^−2^ regime, were used to introduce bone morphogenic protein-2 or transforming growth factor-beta binding peptide sequences to drive hMSC differentiation towards osteogenic or chondrogenic phenotypes, respectively. After 3 weeks of culture in chondrogenic medium, we observed differentiation towards hypertrophic chondrogenic phenotypes with expression of characteristic markers such as collagen X. In osteogenic medium, we observed the upregulation of mineralization markers. In basic media, the chondro-peptide displayed a minor effect on chondrogenesis, whereas the osteo-peptide did not affect osteogenesis. In a subcutaneous rat model, we observed a minimal foreign body response to the constructs, indicating biocompatibility. As proof-of-concept, we finally used a novel AM technology to showcase its potential to create continuous polymer gradients (PCLA and PCLM) across scaffolds. These scaffolds did not display delamination and were mechanically stronger compared to discrete gradient scaffolds. Due to the versatility of the orthogonal chemistry applied, this approach provides a general strategy for the field; we could anchor other tissue specific cues on the clickable groups, making these gradient scaffolds interesting for multiple interfacial tissue applications.

## 1 Introduction

Non-lethal, yet quality of life affecting diseases such as osteoarthritis (OA) are becoming more prevalent due to an aging world population [1]. As a result of low intrinsic healing properties, (small) untreated articular cartilage or osteochondral (OC) lesions tend to progress into end stage OA, ultimately leaving whole joint replacement as the last therapy option [2, 3]. Currently, physicians aim to repair defects at an earlier stage using treatment methods such as microfracture surgery or autologous chondrocyte transplantation [4]. Although these interventions lead to some clinical improvement in the short term (e.g. within 6–12 months from surgery), in the long term they are often unable to restore tissue functions, mainly as a consequence of mechanically inferior collagenous fibrocartilage formation [5–7]. To come up with sustainable solutions for OC regeneration, tissue engineered scaffolds are designed to regenerate damaged hyaline cartilage. However, capturing the full nature of the OC interface in a rationally designed scaffold remains challenging due to both the highly heterogeneous composition as well as anisotropic properties.

The OC unit is often visualized according to a stratified design, in which hyaline cartilage changes step-wise into bone. During the transition chondrocytes change their shape and phenotype, from flattened and persistent (superficial layer) towards bigger and rounded hypertrophic chondrocytes (deep zone), to finally adopt an osteogenic phenotype in the calcified region. The transition in cell type is accompanied by changes in the extracellular matrix (ECM) composition [8]. For example, collagen II is highly expressed towards the joint cavity, but is replaced with mineralization-associated collagen X deposition from the deep zone onward [9]. Due to the cellular and biochemical gradients present in the OC unit, also other gradients arise such as biomechanical and electrical ones [10]. Although the tissue schematically may appear discontinuous, the transition between zones is gradual with exception of the tidemark, delimiting the onset of the calcification [8].

Nowadays, additive manufacturing (AM) technologies are pre-dominantly used to fabricate scaffolds for cartilage and bone applications due to high control over architecture [11]. Due to the presence of gradients in the OC unit, several types have also been introduced across OC scaffolds. For example, changing the porosity or pore shape across scaffolds has been used to influence stem cell fate and ECM production [12–14]. Next to architecture, also biomaterials with intrinsically different properties could be mixed within a construct. Hydrogel precursors have been mixed to generate hydrogel scaffolds containing property gradients, even in a continuous fashion [15–17]. However, hydrogels are generally not suited for high load applications without using an additional supporting framework [18]. Alternatively, biodegradable thermoplastic polymers or composites could be used to generate mechanically stronger AM scaffolds. However, gradually mixing these highly viscous polymer melts during the fabrication process remains challenging, leading to discrete gradient due to interruption of the printing process [19–21]. In addition to poorer biomimicry, these scaffolds are susceptible to delamination due to a mismatch in the mechanical properties. Recently, we developed a new AM technology enabling the extrusion of viscous fillers, such as graphene oxide or hydroxyapatite, into a biomaterial in a continuous fashion [22]. The potential of this technology to create a gradient of inorganic fillers in a polymeric carrier has been clearly demonstrated, but mixing of two thermoplastic polymers has not been explored.

Human mesenchymal stromal cells (hMSCs) are a popular cell source for OC applications, due to their differentiation potential towards both chondrogenic and osteogenic phenotypes. The proteins of the transforming growth factor-beta (TGF-*β*) superfamily play an important role in cartilage and bone development, maintenance and regeneration [23, 24]. To specifically direct hMSCs towards OC phenotypes, TGF-*β*1 or TGF-*β*3 are pre-dominantly used for chondrogenesis and bone morphogenic protein-2 (BMP2) or BMP7 for osteogenesis [25, 26]. Since these proteins are hard to chemically manipulate and have short half-lives *in-vivo*, in many studies the use of TGF-*β* or BMP sequence-mimetic or binding peptides for OC applications is preferred [27]. Most thermoplastic polymers used in melt-extrusion AM are bioinert, and thus incorporating (multiple) bioactive factors can assist hMSC differentiation.

Biologically active groups can be introduced on AM constructs via bulk modification methods on the surface such as aminolysis [28] or polydopamine coatings [29]. If one aims to present biological groups in a spatially controlled manner, the use of non-specific reactive groups, such as amines, is considered challenging. Di Luca *et al* demonstrated that capillary forces can be used to create protein gradients across the scaffolds via aspecifc N-hydroxysuccinimide chemistry [30, 31]. Bio-orthogonal click chemistry is frequently used to specifically and efficiently anchor bioactive groups on biomaterials [32]. Camacho *et al* used thiol-Michael additions to graft differentiation-inducing peptides on low molecular weight poly(*ε*-caprolactone) (PCL). Blends with higher molecular weight PCL were printed sequentially to (co-)present different peptides in bi-layered scaffolds [33]. Guo *et al* anchored azidated tissue specific motives on PCL-alkyne, and fabricated a radial biochemical gradient [34]. These approaches seem promising to guide hMSC fate towards chondrogenic and osteogenic pathways, but the fabricated gradients remain discrete due to usage of multiple print heads. Moreover, the choice of peptide is fixed before manufacturing. Selectively introducing bioactive factors post-fabrication allows for the generation of a versatile platform. When ultimately reactive groups can be (co-)presented on the surface in a gradual manner, the formed neo-tissue may be more continuous.

Here, we report on the fabrication of AM PCL-azide (PCLA) and PCL-maleimide (PCLM) scaffolds with click-type functional groups presented on the fiber surfaces, which can be used for surface modifications with molecules containing complementary groups. We investigated the chondrogenesis and osteogenesis potential *in-vitro* of an azide containing TGF-*β* binding peptide [35, 36] and a thiol containing BMP-2 derived peptide [37], respectively, after 3 weeks of hMSC culture. Moreover, we performed an *in-vivo* subcutaneous rat study to assess biocompatibility of the materials and peptides. Simultaneously, we used a novel AM technology to create continuous gradients of PCLA and PCLM across a scaffold. Although further parameters affecting differentiation *in-vitro* have to be investigated, this print head shows potential for OC application as (bio)chemical gradients can be generated without creating interfaces in an AM scaffold.

## 2 Materials and methods

### 2.1 Materials

PCL (*M*_n_ = 31 kg mol^−1^) was purchased from Sigma Aldrich. All reagents and solvents were purchased from Sigma Aldrich unless stated otherwise, and were used as received. *ε*-caprolactone (TCI chemicals) was dried over calcium hydride and distilled before use. Tin(II)-2-ethyl hexanoate (Sn(oct)_2_) was vacuum distilled prior to use. Thiol modified BMP2-derived (sequence: S-GG-DWIVA, called ‘osteo-peptide’ from here on) and an alkyne modified TGF-*β* binding peptide (sequence: Pra-GG-KGLPLGNSH, called ‘chondro-peptide’ from here on) were purchased from ChinaPeptides (Shanghai, China).

### 2.2 Synthesis

#### 2.2.1 PCLA

Under a dry nitrogen atmosphere, PCL (45 g, 1.45 mmol) was dissolved in anhydrous dimethylformamide (DMF) at a concentration of 150 mg ml^−1^. Subsequently, imidazole (0.068 g, 10 mmol) and p-toluenesulfonyl chloride (1.9 g, 10 mmol) were added and the mixture was stirred overnight at 70 °C. The reaction mixture was concentrated under reduced pressure and the polymer was precipitated in cold methanol (Normapur). The polymer was re-dissolved in chloroform (VWR) and reprecipitated in methanol. This procedure was repeated three more times. The final precipitate was collected as a white solid and dried overnight *in vacuo*. The dried solid (42 g, 1.87 mmol, *M*_n_ = 23 kg mol^−1^) was subsequently dissolved in DMF at a concentration of 150 mg ml^−1^. Then, under a dry nitrogen atmosphere, NaN_3_ (TCI chemicals, 0.61 g, 9.3 mmol) was added and the mixture was stirred for 16 h at 70 °C. The mixture was concentrated under reduced pressure and precipitated in cold methanol. The polymer was re-dissolved in chloroform, precipitated in methanol, and this procedure was repeated three more times. The azide end-functionalized PCL was collected as a white solid and dried *in vacuo*.

Yield: 38 g, 84%, ^1^H nuclear magnetic resonance (NMR) (CDCl_3_, 700 MHz): *δ* (ppm) 4.06 (t, C**H**_**2**_O, *J* = 6.7 Hz), 3.65 (t, C**H**_**2**_OH, *J* = 6.5 Hz), 3.28 (t, C**H**_**2**_N_3_, *J* = 6.8 Hz), 2.31 (t, C**H**_**2**_CO, *J* = 7.5 Hz), 1.65 (m, C**H**_**2**_), 1.39 (m, C**H**_**2**_).

#### 2.2.2 PCLM

In a typical reaction, N-(2-hydroxyethyl)maleimide (169 mg, 1.20 mmol) was loaded into an oven-dried flask and dried *in vacuo* overnight. Under a dry nitrogen atmosphere, *ε*-caprolactone (46.4 ml, 440 mmol) and three drops of Sn(Oct)_2_ (~30 mg, 0.075 mmol) were added and the reaction mixture was stirred for 4 h at 140 °C. Subsequently, the polymer was precipitated in cold diethyl ether. The polymer was re-dissolved in chloroform, precipitated in diethyl ether and this procedure was repeated three more times. The product was collected as a white solid and dried overnight *in vacuo*. Yield: 39 g, 78%, ^1^H NMR (CDCl_3_, 700 MHz): *δ* (ppm) 6.73 (s, C=C**H**, 1H), 4.22 (m, C**H**_**2**_N), 4.06 (t, C**H**_**2**_O, *J* = 6.7 Hz), 3.79 (t, C**H**_**2**_O, *J* = 5.2 Hz), 3.65 (t, C**H**_**2**_OH, *J* = 6.5 Hz), 2.31 (t, C**H**_**2**_CO, *J* = 7.5 Hz), 1.65 (m, C**H**_**2**_), 1.39 (m, C**H**_**2**_).

#### 2.2.3 Click chemistry in solution

The reaction in DMF of the alkyne MegaStokes dye 673 and 7-mercapto-4-methylcoumarin with PCLA and PCLM end groups, respectively, and characterization of the products is described in the supporting information.

#### 2.2.4 Polymer characterization

^1^H NMR and heteronuclear multiple bond correlation (HMBC) spectra were recorded using a Bruker 700 MHz instrument. Attenuated total reflectance Fourier-transformed infrared (FTIR) spectra were recorded on a Bruker instrument in the range 4000–400 cm^−1^. Molecular weights were determined by size exclusion chromatography (SEC) on a Shimadzu system comprising of an autosampler, a Shodex K-G 4A guard (4.6 × 10 mm) column, followed by a Shodex KF-805L (10 *μ*m, 8 × 300 mm) column, a refractive index detector, and a photodiode array detector. Chloroform was used as mobile phase at a flow rate of 1 ml min^−1^ at 40 °C, and using polystyrene standards for molecular weight calibration. Polymer thermal properties were determined by differential scanning calorimetry (DSC) using a TA Instruments Q2000 DSC. Samples were heated from RT to 100 °C at a rate of 10 °C min^−1^ under nitrogen atmosphere, held for 3 min isothermal to erase previous thermal history, cooled to −80 °C at a rate of 10 °C min^−1^, held for 3 min, and finally heated again to 100 °C at 10 °C min^−1^. The melting temperature was determined from the second heating run.

### 2.3 Scaffold fabrication and characterization

#### 2.3.1 Scaffold fabrication

Scaffolds for the *in-vitro* studies were fabricated using a custom developed melt-extrusion based AM technique, which allows mixing of two thermoplastic polymers [22]. A custom-written LabView program was used to control the temperature and pressure during printing. The extrusion screw was operated using a stepper motor with a Trinamic controller board and software. The G-code was generated by the printer software from a GESIM Bioscaffolder 3.0 device. Cylindrically shaped scaffolds were fabricated with the following architecture: a 0–90 pattern, a 550 *μ*m strand distance (center to center), a height of ten layers, and a scaffold diameter of 5.7 mm. To fabricate PCLA or PCLM scaffolds, the polymer was added to both reservoirs, capped by a Teflon plunger, and the full system was heated to 90 °C. The polymer was extruded through a 25G nozzle at a pressure of 5.4 bar and at a screw rotation of 12 RPM. The fiber diameter for all scaffolds was optimized to ~300 *μ*m, by tweaking the translation speed (approximately 400 mm min^−1^ for PCLA and 290 mm min^−1^ for PCLM). A custom-made MATLAB script copied and multiplied the g-code, changing the starting coordinates on the printing platform, to fabricate as many scaffolds as desired in a single fabrication run. For the *in-vivo* study, we used a Bioscaffolder (sysENG, Germany) to manufacture PCLA and PCLM scaffolds with the same geometrical design parameters. The details of the printer settings are described in the supporting information. Scaffolds composed of ten layers of PCLA and PCLM parts were prepared by applying a thin layer of chloroform on one scaffold with a filter paper and pressing the parts together with a tweezer for 10 s.

#### 2.3.2 Gradient scaffold fabrication

Gradient scaffolds of PCLA and PCLM were fabricated with the same architectural parameters as described above, except the height was set to 20 layers. PCLA was added to one reservoir and PCLM to the other, the reservoirs were capped with a plunger, and heated to 85 °C and 90 °C, respectively. The mixing chamber was also heated to 90 °C and the polymer was extruded through a 25 G nozzle with a screw speed of 12 RPM. The Matlab script was used again to produce more scaffolds, either 6 or 17, simultaneously. The g-code generated from the script finishes all first layers of the scaffolds and proceeded with this layer-by-layer fabrication. First, a pressure of 5.4 bar was applied to the reservoir containing PCLA. After either two or six layers, the pressure was removed from this reservoir and a pressure of 5.4 bar was applied to the PCLM reservoir. The gradient distribution and location along the *y*-axis were controlled by the timing of the pressure switch or the number of manufactured scaffolds (equivalent to the amount of deposited material) in a single run. In the latter approach, more material was deposited to finish all the layers of a certain height, leading to a narrow distribution of the gradient in a singular scaffold.

The polymer composition of the top and bottom layers of the fabricated scaffolds were analyzed by ^1^H NMR spectroscopy. Moreover, to macroscopically visualize the material switch, 0.002% w/w fluorescent red dye (MacroLex®), relatively to the amount of PCLM, was used. To this end, the dye was added to a solution of PCLM in chloroform (150 mg ml^−1^) and subsequent evaporation of the solvent *in vacuo*. Gradient scaffolds were prepared using the same printer settings as described above.

#### 2.3.3 Scanning electron microscopy

The morphology of the scaffolds was evaluated using a Jeol JSM-IT200 InTouchScope (Tokyo, Japan) SEM at an accelerating voltage of 10.0 kV and a working distance of 10 mm. Before analysis, the scaffolds were gold coated using a Cressington sputter coater 108 Auto set at 30 mA for 60 s.

#### 2.3.4 Mechanical testing

The mechanical properties of the scaffolds were determined by compression testing using a TA ElectroForce system (TA instruments) equipped with a 450 N load cell at ambient conditions. The instrument was controlled with Wint7 software. After a preload of 10 N, tests were conducted at a strain rate of 0.01 mm s^−1^ up to a maximum of 50% compression or reaching the loading cell limit. PCLA, PCLM, merged and gradient (PCLA + PCLM) scaffolds were tested. Continuous gradient scaffolds were manufactured by printing 17 scaffolds in a single run and applying a pressure switch after layer 6. Prior to testing, the height and diameter of the samples were measured with a caliper. The compressive modulus was calculated from the steepest slope of the stress–strain curve in the linear elastic region between approximately 4-7% strain. The strain at break was set at the point where the elastic slope decreased by 1%.

### 2.4 Surface functionalization

#### 2.4.1 Coupling dyes on the fiber surface

PCLA scaffolds (~42 mg, 3.36 × 10^−3^ mmol azide end-groups) were separately placed in 15 ml centrifugation tubes containing 1.5 ml of water, Cu(II)SO_4_ · 5H_2_O (0.26 mg, 1.05 × 10^−3^ mmol), and sodium ascorbate (2.08 mg, 1.05 × 10^−2^ mmol). Finally, 0.98, 0.098, or 0.0098 mg (0.3, 0.03, or 0.003 equiv. relative to the amount of azide groups in the bulk of the scaffold) of the alkyne MegaStokes dye was added and the tubes were left on a roller bank for 22 h at room temperature (RT).

PCLM scaffolds (~37 mg, 1.54 × 10^−3^ mmol maleimide groups) were separately placed in 15 ml centrifugation tubes containing 2 ml of buffer (0.1 M monosodium phosphate, 0.15 M sodium chloride, 10 mM ethylenediaminetetraacetic acid, pH 7.2) containing 1% v/v triethylamine and 0.25% w/v Tris(2-carboxyethyl)phosphine hydrochloride. Finally, 2.90 mg, 0.29 mg, or 0.029 mg (0.86, 0.086, or 0.0086 equiv. relative to the amount of PCLM polymer in the scaffold) of a thiol containing dye (Sun Fluor 488 thiol, Nanocs Inc., USA) was added and the tubes were left on a roller bank for 21 h at RT.

Subsequently, all scaffolds were thoroughly washed with dimethylsulfoxide, water, ethanol, and finally dried *in vacuo*. The scaffolds were visualized using a Nikon TI-E epi-fluorescent microscope and analyzed with Fiji software for their fluorescence intensity on the fibers. To determine the surface density of coupled dye, spectrophotometric evaluation was performed using an Agilent Cary Eclipse Fluorescence spectrophotometer. Scaffold samples were dissolved in chloroform at a concentration of 30 mg ml^−1^, placed in quartz cuvettes, and measured with a high photomultiplier tube voltage (600 V) with an excitation and emission slit width of 10 nm and 5 nm, respectively. Excitation wavelengths of the alkyne and thiol dye were set at 550 nm and 495 nm, respectively. The intensity at the emission wavelength of 642 nm and 525 nm, respectively, was used to calculate the surface coupled dye concentration of the samples. Standard curves were prepared from dye solutions in chloroform.

#### 2.4.2 Coupling peptides on the fiber surface

If scaffolds were used for *in-vitro* or *in-vivo* purposes, they were first sterilized with 70% ethanol or 2 h UV sterilization, respectively. We used similar reaction conditions as described above using sterile filtered solutions and under sterile conditions. PCLA scaffolds were separately placed in 15 ml centrifugation tubes, containing 0.87 ml of water, 1.70 mg of TGF-*β* derived peptide, 0.37 mg of Cu(II)SO_4_ · 5H_2_O, and 2.97 mg of sodium ascorbate and left on a shaker for 19 h at RT. PCLM scaffolds were separately placed in 15 ml centrifugation tubes containing 0.87 ml of coupling buffer containing 1% v/v triethylamine, 0.25% w/v Tris(2-carboxyethyl)phosphine hydrochloride, and 1.44 mg osteo-peptide and left on a shaker for 19 h at RT. All scaffolds were extensively washed with phosphate buffered saline (PBS) before further use. The static water contact angles of dried scaffolds were measured by the sessile drop method on a Kruss Contact Angle Measuring System and using Drop Shape Analysis 4 software.

### 2.5 Biological assays

#### 2.5.1 Cell expansion

Human bone marrow derived hMSCs were isolated by Texas A&M Health Science Center from a male 22 year old donor [38]. Briefly, aspirated bone marrow was centrifuged to isolate mononuclear cells, and the hMSC differentiation potential was verified. The hMSCs were further expanded and tested for their differentiation potential. Cells were received at P1, plated at a density of 1000 cells cm^−2^ in tissue culture flasks, and expanded in cell culture media consisting of alpha miminum essential medium with Glutamax and no nucleoside (Gibco), supplemented with 10% v/v fetal bovine serum (Sigma) at 37 °C and 5% CO_2_ until a maximum of 80% confluence was reached. Cells at passage 5 were used for experiments.

#### 2.5.2 Cell seeding

Scaffolds, PCLA and PCLM, were sterilized in 70% ethanol overnight and washed with PBS. Part of the scaffolds were sterile functionalized with peptides as described in section 2.4.2. The scaffolds were dried on sterilized filter paper and then placed in nontreated hydrophobic tissue culture well-plates. A cell suspension was prepared in basic media (BM = culture media containing also penicillin (100 U ml), streptomycin (100 *μ*g ml)) and seeded as a drop of 150.000 cells on top of each scaffold. Cells were left to attach for 2 h in an incubator at 37 °C and 5% CO_2_. Thereafter, the scaffolds were flipped and left for another 2 h to obtain a homogeneous cell distribution. After 4 h, 1.5 ml of BM was carefully added to the wells containing the scaffolds. The next day, the scaffolds were placed in fresh wells plates. Besides scaffolds, hMSCs were seeded in BM cell culture treated well-plates at a density of 1.2 × 10^4^ cells cm^−2^. In a preliminary study, we also seeded hMSC in dextransupplemented media [39]. The details are described in the supporting information.

#### 2.5.3 Cell differentiation

After 24 h culturing in BM, the differentiation process was initiated. PCLA (− and + chondropeptide) constructs were cultured in BM and chondrogenic medium (CM). CM consisted of high glucose (4.5 mg ml^−1^) Dubelcco’s modified Eagle medium with 100 *μ*g ml^−1^ sodium pyruvate (Gibco), 200 *μ*M ascorbate-2-phosphate (ASAP), 1% 100× insulin–transferrin–selenium liquid media supplement (Thermo Fisher Scientific), 40 *μ*g ml^−1^ proline, penicillin (100 U ml^−1^), and streptomycin (100 *μ*g ml^−1^). The media was completed by freshly supplementing 100 nM dexamethasone and 10 ng ml^−1^ of TGF-*β*3 before use. PCLM (− and + osteo-peptide) constructs and 2D plated cells were cultured in BM and osteogenic medium (OM), which is BM supplemented with 100 *μ*M ASAP, 10 nM dexamethasone, and 10 mM *β*-glycerophosphate. The differentiation process was continued up to 21 d with media changes every 2–3 d. The culturing of hMSC pellets and biological analysis is described in the supporting information.

#### 2.5.4 Biochemical assays

##### 2.5.4.1 Alkaline phosphatase (ALP) activity

As an osteogenic differentiation marker, the ALP activity in osteogenic (PCLM) constructs cultured in BM and OM was determined after 21 days. Samples were washed twice with PBS and stored at −80 °C. The scaffolds were cut in halves, and all samples were freeze-thawed three times. Then, cell lysis buffer (0.1 M potassium phosphate, 0.1 M dibasic potassium phosphate, 0.1% v/v Triton X-100, pH 7.8) was added and the constructs were incubated for 1 h at RT. To 10 *μ*l of the cell lysate, 40 *μ*l of the chemiluminescent substrate CPD-star® (ready-to-use, Roche) was added. After 15 min of incubation, luminescence (*λ*_em_ = 470 nm) was measured using a spectrophotometer (CLARIOstar®, BMG Labtech). Values determined were normalized against DNA content. The remaining cell lysate was used for DNA and hydroxyproline (HYP) assays.

##### 2.5.4.2 DNA assay

A DNA assay was performed using the CyQuant cell proliferation Assay Kit (Thermo Fisher Scientific) after 24 h or at day 21. Chondrogenic (PCLA) constructs cultured in BM and CM were first washed two times with PBS, stored at −80 °C, cut in halves, and freeze-thawed three times. Consecutively, all constructs (chondrogenic and pre-digested osteogenic from section 2.5.4.1) were submerged in Proteinase K lysis buffer (1 mg ml^−1^ in Tris/EDTA pH 7.6) overnight at 52 °C. The lysis buffer was added in a one-to-one volume ratio to the osteogenic constructs. Next, all samples were again freeze-thawed three times. Volumes of digested samples were collected and stored for glycosaminoglycan (GAG) and HYP assays. The remaining volume was incubated in a one-to-one volume ratio with lysis buffer (20× diluted cell lysis buffer from the kit) containing RNase A (1:1000) to degrade cellular RNA for 1 h at RT. According to the manufacturer’s protocol, the lysed samples were incubated with the fluorescent dye for 15 min and fluorescence was measured at *λ*_ex_ = 480 nm and *λ*_em_ = 520 nm using a CLARIOstar®. DNA concentrations were determined using a standard curve prepared from a series of DNA solutions.

##### 2.5.4.3 GAG assay

As a chondrogenic differentiation marker, the production of GAGs on digested samples of chondrogenic scaffolds was determined. Samples from the proteinase K digestion step (see section 2.5.4.2) were used to measure the GAG content using a 1,9-dimethyl-methylene blue zinc chloride double salt (DMMB) solution (16 mg DMMB in 5 ml of ethanol). In brief, 150 *μ*l of DMMB solution was mixed with 25 *μ*l of sample and 5 *μ*l 2.3 M sodium chloride in a black well plate. The difference in absorbance between 525 and 595 nm was measured using a CLARIOstar®. The GAG content was determined using a standard curve prepared from chondroitin sulfate solutions (from shark cartilage) and normalized to the amount of DNA.

##### 2.5.4.4 HYP assay

Proteinase K digested samples (see section 2.5.4.2) were diluted in Tris/ethylenediaminetetraacetic acid buffer and hydrolyzed by the addition of 37% HCl in a one-to-one volume ratio for 18 h at 110 °C. The solvent was evaporated in a vented oven. Quantification was done by first oxidizing the HYP in a chloramine T containing buffer for 20 min at RT. The product was made fluorescent by reaction with 4-dimethylaminobenzaldehyde. The absorbance of the solution was measured at 570 nm. A standard curve was prepared from a series of trans-4-hydroxy-L-proline solutions subjected to the same process as described above. The total collagen content was calculated using a HYP to collagen ratio of 1:7.69. Determined values were normalized against DNA content.

#### 2.5.5 Real-time polymerase chain reaction (RT-PCR)

Both chondrogenic and osteogenic constructs were minced, collected in 1 ml of Trizol (Invitrogen), and stored at −80 °C. RNA was extracted with the RNeasy Mini kit with on-column DNase treatment (Qiagen, Hilden, Germany) according to the manufacturer’s protocol. cDNA was synthesized from 140 ng total RNA, using an iScript cDNA synthesis kit (Bio-Rad, California, USA) following manufacturer’s instructions. RT-PCRs were prepared in a total volume of 20 *μ*l with 10 *μ*l iQ SYBR green Supermix (Bio-Rad), 1.25 *μ*M forward and reverse primers (table S1), 5 ng of cDNA, and nuclease-free water. A CFX96^TM^ IVD RT-PCR system (Bio-Rad) was used with a thermal cycle of 95 °C for 3 min, 95 °C for 15 s and 55 °C for 30 s for a total of 40 cycles. Ct values of RT-PCR were normalized against the housekeeping gene (Glyceraldehyde 3-phosphate dehydrogenase) and analyzed using the ΔΔCt model [40].

#### 2.5.6 Immunofluorescence

Both chondrogenic and osteogenic samples were fixed after 7 days or 21 days in 4% paraformaldehyde (PFA), cut open vertically, washed with PBS, and cells were permeabilized for 20 min in a 0.1% v/v Triton X-100 solution in PBS. After rinsing, a solution of 3% v/v bovine serum albumin (BSA) and 0.01% Triton-X100 in PBS was used for blocking. After removing the blocking solution, samples were incubated overnight at 4 °C with rabbit anti-RUNX2 (1:250, ab192256), mouse anti-SOX9 (1:200, ab76997), mouse anti-collagen I (1:300, ab6308), rabbit anti-collagen II (1:400, ab34712), mouse anti-collagen X (1:200, ab49945), rabbit anti-osteocalcin (OCN) (1:250, ab93876), or rabbit anti-osteopontin (OPN) (1:400, ab8448) antibodies, and AlexaFluor Phalloidin (1:100, either Phalloidin-488 or 568). Subsequently, the samples were rinsed with 0.3% v/v BSA and 0.01% Triton-X-100 in PBS. The samples were incubated with goat anti-rabbit and goat anti-mouse secondary antibodies for 45 min at RT (1:200, either Alexa Fluor 488, 568, or 647) and shielded from light. After washing, nuclei were counterstained with DAPI (0.233 *μ*g ml^−1^) for 15 min. Samples were imaged using a Leica TCS SP8 CARS confocal microscope.

#### 2.5.7 Further biological evaluations

Histological evaluation of the constructs with Alizarin red S and safranin-O staining, and a collagen X evaluation of the supernatant is described in the supporting information.

### 2.6 *In-vivo* procedure

#### 2.6.1 Study design and preparation

All rats (Crl:NIH-Foxn1rnu, Charles River; female; 8–10 weeks old; weight range, 140–212 g) received over 1 week acclimatization in the animal research facility in Maastricht University before surgery. The animals were kept in a climate controlled environment (21 °C, 12 h light/dark cycles) and had ad libitum access to food (10 mm Sniff rat/mouse sterilized food in pellets) and water. The cylinder-shaped scaffolds were fabricated as described in the supporting information. We implanted bare scaffolds without or with coupled peptides, according to the procedure in section 2.4.2. Both of these scaffold types were also implanted with pre-seeded hMSC, according to the procedure in section 2.5.2, using 200.000 cells per scaffold. The constructs were implanted subcutaneously for 3 and 6 weeks, after which the animals were sacrificed.

#### 2.6.2 Surgical procedure

Before surgery, buprenorphine (0.05 mg kg^−1^ bodyweight) and carprofen (4 mg kg^−1^) were administered to the rats. Induction of anesthesia for animals was applied with 3%–4% (v/v) isoflurane, mixed with oxygen and air. Anesthesia was maintained by 2% (v/v) isoflurane, and adjusted if clinical behavior of the animal changed during surgery. After shaving and sterilization of the implant site, four 1 cm long linear incisions parallel to the spine were made on each side. Four subcutaneous pockets of maximum 10 mm × 10 mm were created with the help of scissors, after which the scaffolds were implanted randomly. The skin was closed intracutaneously by using an absorbable suture (Monocryl 4-0) and analgesics (0.03 mg kg^−1^ buprenorphine) were infused 8 h after surgery. On day 1 and 2 after the operation, each animal received a dose of carprofen (4 mg kg^−1^ bodyweight). The animals were evaluated daily for 3 or 6 weeks with a welfare logbook score. The animals were sacrificed with CO_2_ overdose.

#### 2.6.3 Tissue processing and evaluation

The pockets with the samples and the surrounding tissue were collected from the animals for histological analysis and kept in PBS. The pockets were then carefully cut to a size of 2 × 2 cm. Samples were fixed for 24 h at 4 °C in a 4% PFA solution in tris-buffered saline solution. Subsequently, the samples were transferred to solutions of 30% (w/v) sucrose, one-to-one volume ratio of 30% sucrose and optimal cutting temperature (OCT, Scigen), and OCT, for 24 h each, at 4 °C, to allow good tissue penetration of OCT. Subsequently, samples were placed in perpendicular direction (i.e. the cross-section is facing the surface) into peel-A-way® histology molds of 22 × 22 × 20 mm. Samples were then frozen on the liquid–vapor interface of a liquid nitrogen tank to avoid bubble formation, and stored at −30 °C until further use. Sections of 7 *μ*m were cut using a Leica 3050S cryrotome and recovered with the use of cryofilm (section-lab Co, Ltd, Japan). The cryofilm was finally attached to a glass slide by the extremes with double sided cryotape (3M^™^ 9088) and kept at −30 °C until analysis. Sections were stained with hematoxylin and eosin (H&E) and Masson’s trichrome (MT) staining. For H&E, sections were rehydrated, stained with Gill’s hematoxylin for 5 min and counterstained with alcoholic Eosin Y for 1 min. For MT staining (HT15, Sigma), the manufacturer’s protocol was used. Briefly, rehydrated sections were fixed in Bouin’s solution for 1 h, followed by a rinsing step with tap water. Sections were then stained with Weigert’s iron hematoxylin working solution for 5 min, washed with tap water, stained with Biebrich scarlet-acid fuchsin solution for 5 min and washed again in tap water. Afterwards, sections were allowed to differentiate in phosphomolybdic–phosphotungstic acid solution for 5 min, followed by incubation in aniline blue solution for 5 min and a quick wash in 1% acetic acid solution. Images were taken using a stereomicroscope (Nikon SMZ800 with Q-imaging Retiga 1300 camera).

### 2.7 Statistical analysis

All quantitative data are presented as means ± standard deviation. Statistical analysis was performed using GraphPad Prism. When a Shapiro–Wilk test confirmed a normal distribution of the data, we determined statistical significance by a one-way analysis of variance with Tukey’s honestly significant difference multiple comparison tests. Otherwise, a non-parametric test was performed; (****) *p <* 0.0001, (***) *p <* 0.001, (**) *p <* 0.01, (*) *p <* 0.05.

## 3 Results and discussion

**Figure 1:**
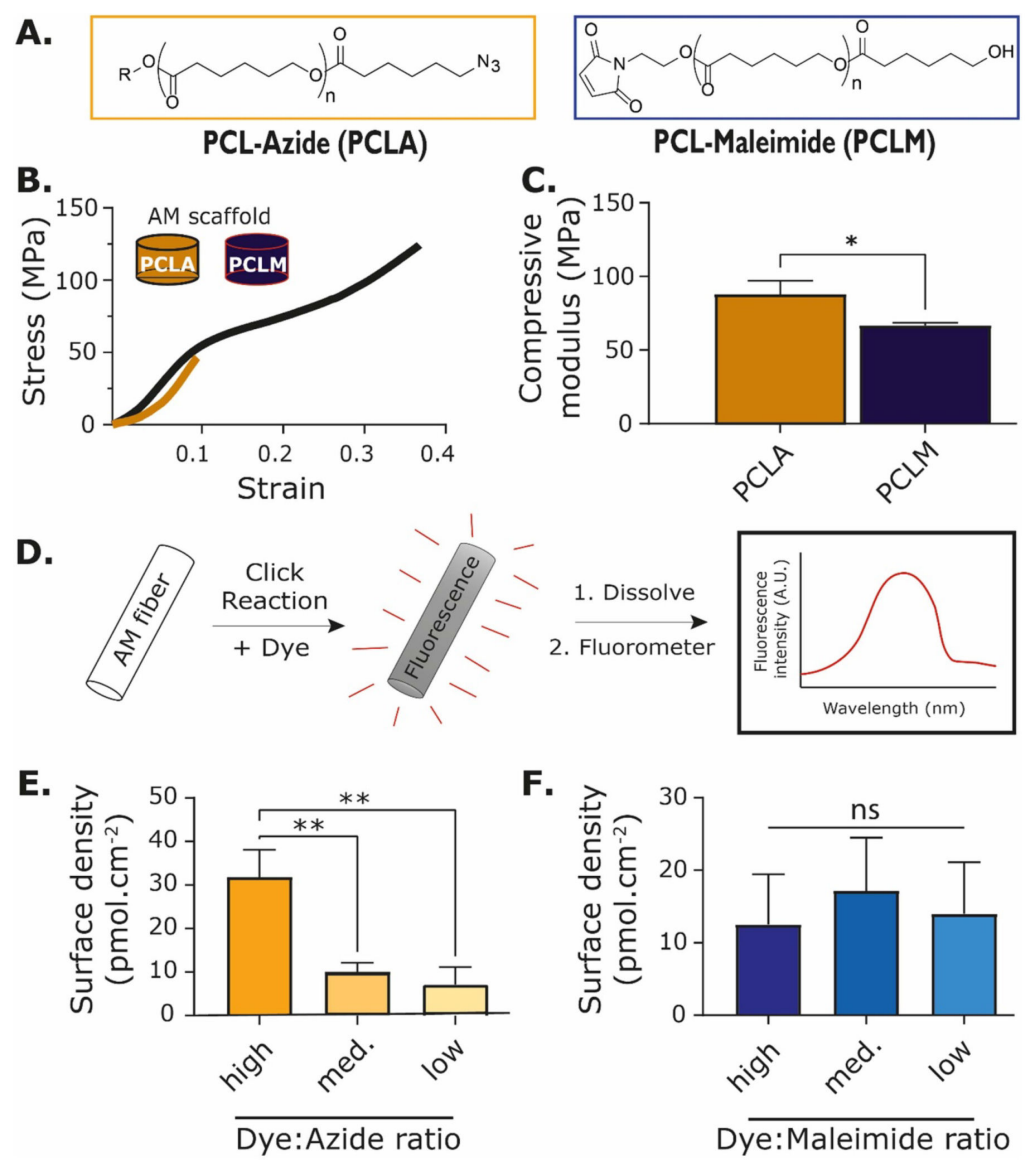
Applied materials and surface modification with complementary fluorescent dyes. (A) PCL containing a terminal azide (PCLA) or maleimide (PCLM) group. (B) Representative stress–strain curves. All curves can be found in the supporting information (figures S6(A) and (B)). (C) The average compressive modulus of PCLA and PCLM scaffolds. (D) Click chemistry was applied to attach dyes to the fiber surfaces, followed by spectrofluorimetric analysis of dissolved scaffolds. (E) and (F) Surface concentration of the dye in PCLA and PCLM scaffolds, respectively. The stoichiometric excess of dye relatively to the total moles of functional groups in the bulk of the scaffold increased an order of magnitude, from low to high, *N* = 3. Data is presented as mean *±* SD, *p < 0.05, **p < 0.01, ns = non-significant, Med. = medium.

To fabricate AM scaffolds that could guide hMSC differentiation into either a chondrogenic or osteogenic pathway, we selected two differentiation-inducing peptides for coupling onto the fiber surfaces. Ultimately, we aim to attach these peptides simultaneously on one scaffold. Since it was demonstrated that different types of click chemistry were chemically compatible (orthogonally reactive), we considered using functional groups from this toolbox to react peptides onto a scaffold in a chemoselective manner [41]. Moreover, these orthogonal reactions have been already applied at relatively low reaction concentrations such as intracellular imaging [42], bio-conjugation [43, 44], and surface functionalization [45]. In this study, we introduced terminal azide and maleimide groups on PCL (figure 1(A)). Molecules or peptides containing an alkyne or thiol functional group were used to perform copper(I)-catalyzed alkyne-azide or thiol-Michael reactions on AM fibers, respectively.

### 3.1 Material synthesis

PCLA was synthesized by conversion of PCL hydroxyl end groups via a chlorination-azidation pathway (scheme S1(A)) [46]. The degree of substitution was determined by assessing the shifted terminal methylene protons adjacent to the azide group by ^1^H NMR, which was about 65% (figures S1(A) and (B)). The presence of the azide group was confirmed by FTIR and ^1^H–^15^N HMBC spectroscopy (figures S2 and S3). Based on NMR end group and SEC analysis a decrease in molecular weight from 31 to 23 kg mol^−1^ was observed (figures S1(B) and S4, respectively). This decrease was likely due to the applied basic reaction conditions that resulted in partial hydrolysis of the ester groups. PCLM was synthesized by ring opening polymerization of *ε*-caprolactone using N-(2-hydroxyethyl)maleimide as an initiator and Sn(oct)_2_ as a catalyst (scheme S1(B)). The molecular weight of the PCLM was 21 kg mol^−1^ according to NMR analysis and the degree of functionalization was approximately 70% (figure S1(C)). The SEC analysis confirmed that PCLA had a similar molecular weight as the elution time was similar. Both polymers showed a thermal behavior similar to pristine PCL, with a *T*_m_ of approximately 56 °C (figure S5). Notably, the melting enthalpy of the PCLA (67.6 J g^−1^) was somewhat higher than that of the PCLM (60.6 J g^−1^), indicating a higher crystallinity. The similar melting temperature and molecular weight of PCLA and PCLM suggested comparable printability like unfunctionalized PCL.

### 3.2 AM scaffold characterization

#### 3.2.1 Mechanical characterization

Subsequently, we manufactured up to 35 cylindrically shaped AM scaffolds simultaneously from either PCLA or PCLM. All scaffolds were designed with the same printing pattern to exclude effects on mechanical properties (figures S7(A) and (B)). The scaffolds had a fiber diameter, pore size, and layer height ranging from 280 to 289 *μ*m, 66.6 × 10^3^ to 68.4 × 10^3^ *μ*m^2^, and 254 to 279 *μ*m, respectively (table S2). The scaffolds were subjected to a compressive test and representative stress strain curves are depicted in figure 1(B). Both scaffolds displayed an initial elastic response up to approximately 10% strain. The compressive modulus of PCLA scaffolds (88 ± 9.1 MPa) was higher than PCLM scaffolds (67 ± 1.8 MPa). After the elastic region, a decrease in the slope was observed in the PCLM, associated to collapsing pores, followed by an exponential increase as a result of densification. PCLA scaffolds crumbled after the elastic region and displayed a strain-at-break of 0.101 ± 0.015. PCLM scaffolds did not break and displayed a yield strain of 0.093 ± 0.009. We attributed the different modulus and mechanical behavior to the higher crystallinity of PCLA (figure S5).

#### 3.2.2 Surface modification

To evaluate the availability of reactive functional groups on the surface of the fibers after extrusion, we reacted the PCLA and PCLM scaffolds with complementary molecules. Because quantitative analysis of surface peptide groups would be difficult, alkynated and thiolated fluorescent dyes were used as a model. An initial experiment was conducted with the polymers dissolved in solution (DMF) to confirm the efficient reactivity of the dyes to the parent PCLA and PCLM (figure S8). We also confirmed with NMR that the present functional groups were able to resist the elevated temperatures required during the printing process (figure S9).

Subsequently, we anchored alkynated and thiolated fluorescent dyes to the AM scaffolds for spectrofluorimetric analysis as depicted in figure 1(D). After dissolution of the scaffolds, the surface concentration of dye attached onto the scaffolds was determined by using a calibration curve. By approximating the surface area of the scaffolds (average mass of the scaffolds and a fiber thickness of 300 *μ*m), the surface density of the functional groups was calculated (tables S3 and S4). For the PCLA scaffolds, we found an increase in the surface density from 7.1 ± 4.0 to 32 ± 6.3 pmol cm^−2^, when the molar ratio of alkyne (dye) to the azide (in the bulk) groups was increased from 3.3 to 333 (figure 1(E)). The control for aspecific absorption was an order of magnitude lower, confirming successful washing (table S3). Reacting the thiolated dye with the PCLM scaffolds afforded surface densities in between 13 ± 7.1 and 20 ± 6.9 pmol cm^−2^, with no specific trend (figure 1(F)). In absence of a base, we found a similar density of the dye on the surface, showing that the thiol-Michael addition can also rapidly occur at physiological pH [47]. Since untreated PCLM scaffolds displayed lower (auto)fluorescent signal than all other samples after extensive washing, we concluded that the surface was successfully modified. These observations indicated that we had a successful reaction of the dye on the surface instead of absorption.

Overall, the PCLM surface seemed to reach saturation, whereas the PCLA surfaces displayed a decreasing trend based on the stoichiometric excess addition of the dye. Before dissolution for spectro-fluorimetric analysis, we visualized the (un)treated scaffolds under the fluorescent microscope to assess the intensity and homogeneity of the signal from the dye. The mean gray values also indicated signal saturation for the PCLM scaffolds, whereas a similar increasing trend as the surface density was observed for the PCLA scaffolds. Moreover, the relatively small standard deviation implied a homogenous functionalization of the fibers (figure S10). The aligned trends between the fluorescence intensity and spectrofluorimetric analysis were a further confirmation of the surface density results. A possible explanation for the different surface density values between PCLA and PCLM scaffolds could be related to the nature of the reactive groups. The aqueous conditions applied in the coupling of the dyes to the scaffolds might lead to a slightly higher surface availability of the more hydrophilic azide groups. Finally, we reacted the differentiation-inducing peptides on the corresponding PCLA and PCLM scaffolds using the highest stoichiometric excess of peptide. WCA experiments revealed fast absorption of the droplet in contrast to the unmodified scaffolds (figure S11). The enhanced hydrophilicity indicated a successful postmodification with peptides. Because the click reactions of peptides to the surface were conducted using the same conditions as used for the dyes we assume that the surface density of the peptides is also approximately 20 pmol cm^−2^.

The use of click-type chemistry is a straightforward method to introduce bioactive groups on the surface of scaffolds to guide cell behavior. Biologically active molecules often act in a concentrationdependent manner, thus quantitative analysis on surface density is important. However, re-designing Food and Drug Administration-approved thermoplastic polymers with reactive groups is not commonly performed. The Becker group had a pioneering role in synthesizing copolyesters containing clickable groups for stereolithography applications [48]. Li *et al* modified a poly(ester urea) polymer with propargyl groups to introduce both complementary dyes as well as osteogenic peptides on a melt-extruded AM scaffold. The 2% of the repeating units contained a propargyl group, leading to a surface density of 75 ± 5 pmol cm^−2^ of the alkyne group on the AM fibers [49]. In the PCLA and PCLM polymers, approximately 0.5% of the repeating units of each polymer chain contained a reactive group. Thus, a slightly lower surface density was expected, and aligns with their work.

### 3.3 *In-vitro* hMSC study

**Figure 2:**
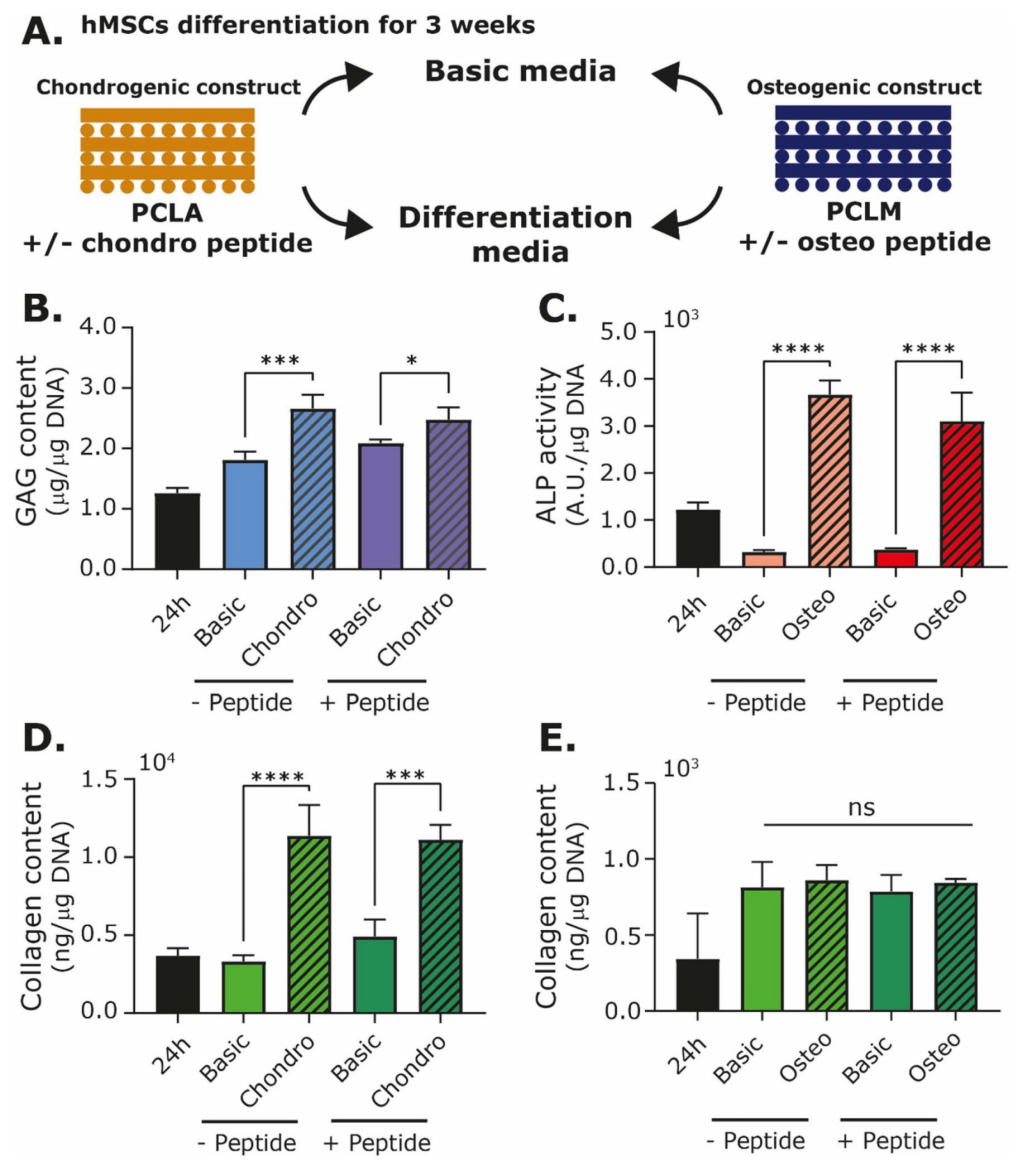
Classic differentiation markers deposited after 21 days. (A) Schematic of the hMSC differentiation study. The graphs on the left and right side of the figure correspond to the chondrogenic and osteogenic constructs, respectively. (B) Normalized GAG content against DNA content. (C) Normalized ALP activity against DNA content. (D) and (E) Normalized collagen production against DNA content, *N* = 3. Data is presented as mean *±* SD, ns = non-significant, *p < 0.05, **p < 0.01, ***p < 0.001, ****p < 0.0001.

The cell proliferation, distribution, and differentiation of hMSCs on PCLA and PCLM scaffolds with or without surface coupled peptides were studied. Cells were cultured in either BM, OM or CM differentiation media for 21 days (figure 2(A)).

#### 3.3.1 Cell adhesion and proliferation

After 21 days of culture, we assessed hMSC proliferation and distribution in the constructs. We observed higher proliferation rates in BM and OM, whereas in CM a decrease in cell number as compared to the initial seeding values was found (figure S12). Lowered proliferation rates are typically observed in hypertrophic chondrocytes, and enhanced rates during osteoblast maturation [50, 51]. The addition of the peptide in both BM and differentiation media did not significantly affect cell proliferation. Upon evaluation of the distribution of the cells throughout the scaffolds, we observed cells sinking towards the bottom, even when we flipped the scaffolds on a weekly basis (figure S13). Most thermoplastic polymers in AM are hydrophobic and do not allow for fast protein absorption, leading to anisotropic distribution of the cells [13].

We tested simultaneously the capability of dextran added to the culture media as viscosity enhancer as described by Camara-Torres *et al* [39]. The enhanced media viscosity resulted in an improved cellular distribution already after 7 d (figure S14(A)). The dextran supplementation method circumvents cell sinking, but could potentially mask the peptides or translate into macromolecular crowding effects in the OC lineage [52], affecting the cellular outcome. For example, it might affect cell proliferation rates since we observed a doubling on cell number after 7 days when cells were cultured in BM containing ASAP, but only after 21 days when cells where seeded in the absence of dextran (figure S14(B)). In articular cartilage, the cell number is gradually reduced from the superficial towards the calcified layer [8]. Thus, we decided to proceed our study by seeding the cells in a droplet on top of the scaffolds, relying on capillary and gravitational forces for penetration.

#### 3.3.2 Cell differentiation

##### 3.3.2.1 Chondrogenic differentiation

Depending on their location in the articular cartilage, chondrocytes have different morphologies corresponding to their phenotype and ECM deposition, depending on their location in the tissue. Thus, it is important to verify the phenotype of differentiated hMSCs and the matrix deposited within the formed neo-cartilage in response to the construct material (PCLA) and the chondro-peptide. To this end, we investigated characteristic differentiation markers such as various collagen types or GAGs, from the genetic to the protein level, after 21 days in BM or CM.

Culturing hMSCs in PCLA scaffolds in CM showed a significant increase in the deposition of GAGs compared to cultures in BM. Cells cultured in PCLA scaffolds without and with coupled peptide in CM deposited similar values of 2.66 ± 0.23 and 2.48 ± 0.20 *μ*g GAG/*μ*g DNA, respectively. In BM, these values were 1.81 ± 0.14 *μ*g GAG/*μ*g DNA for constructs without peptide and 2.09 ± 0.06 *μ*g GAG/*μ*g DNA with coupled peptide (figure 2(B)). Comparing the lowest GAG deposition in the PCLA scaffold in BM with the maximum deposition in the CM, we observed an increase of 0.85 *μ*g GAG/*μ*g DNA. In response to the peptide, an increasing trend of 0.28 *μ*g GAG/*μ*g DNA was displayed in the BM. Assuming that 0.85 *μ*g GAG/*μ*g DNA is the maximum enhancement in GAG deposition, 0.28 *μ*g GAG/*μ*g DNA corresponds to a 39% increase in response the peptide alone in BM. The increase in GAG deposition did not lead to appreciable visual differences when samples were stained with safranin-O (figure S19).

**Figure 3:**
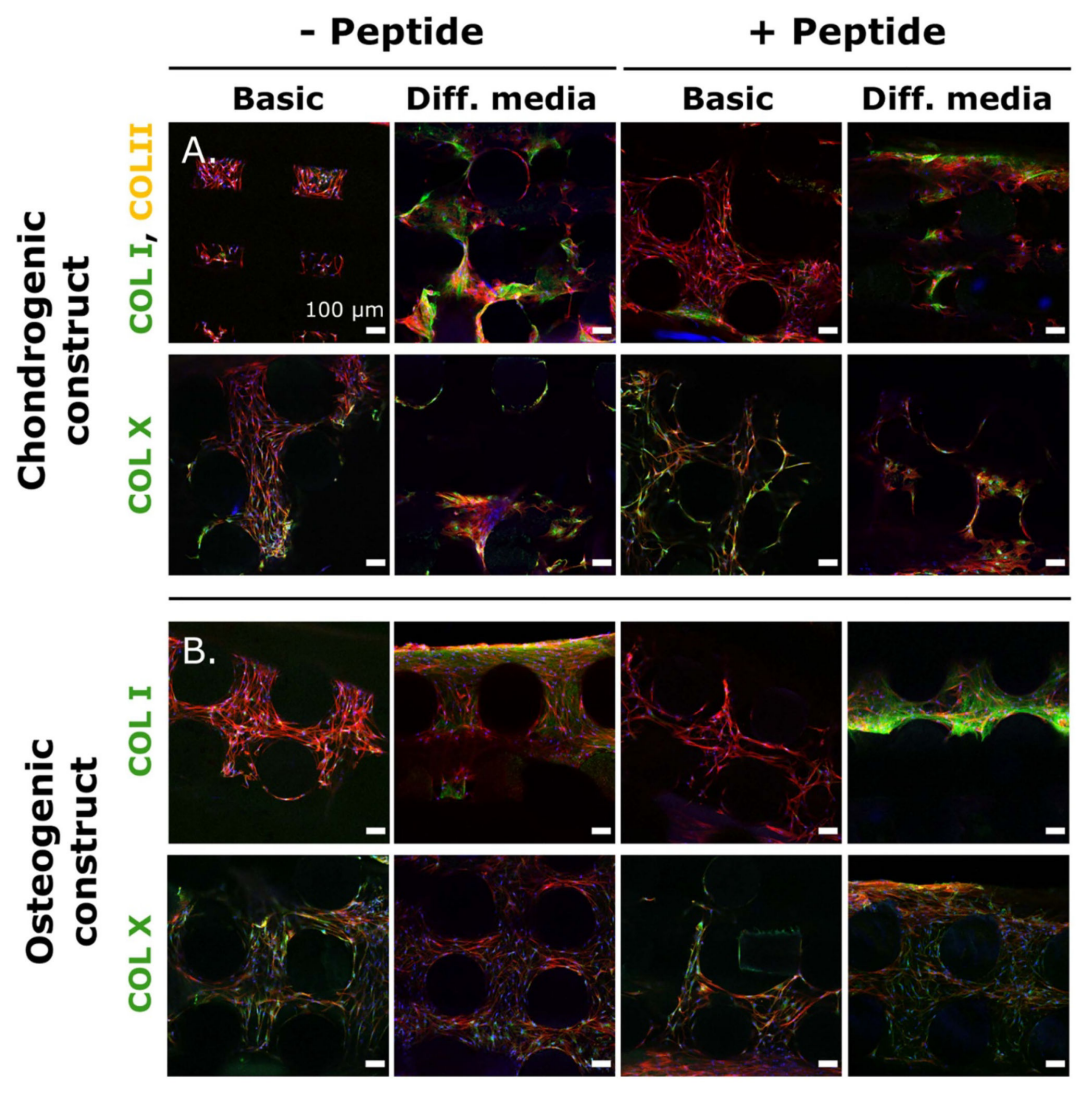
Collagen deposition of hMSC after 21 days of differentiation. All samples were stained for nuclei (blue, DAPI) and actin (red, phalloidin). In panel (A), chondrogenic constructs were stained for deposition of collagen I (green, top row), collagen II (yellow, top row) and collagen X (green, bottom row). The split channel images of the chondrogenic constructs are found in collagen I (green, top row) and collagen X (green, bottom row). The split channel images of the osteogenic constructs are found in figure S15 and the 25× magnification images in figure S16. In panel (B), osteogenic constructs were stained for deposition of collagen I (green, top row) and collagen X (green, bottom row). The split channel images of the osteogenic constructs are found in figure S17 and the 25× magnification images in figure S18. Scale bar = 100 *μ*m, N = 2.

Similar trends were observed for the total collagen deposition upon incubating the constructs in CM (without peptide: 1.1 × 10^4^ ± 2.0 × 10^3^ vs with peptide: 1.1 × 10^4^ ± 0.9 × 10^3^ ng collagen/*μ*g DNA) and BM (without peptide: 3.3 × 10^3^ ± 0.4 × 10^3^ vs with peptide: 4.9 × 10^3^ ± 1.1 × 10^3^ ng collagen/*μ*g DNA) (figure 2(D)). Comparing the deposition of collagen in response to the peptide in BM to the maximum deposition as result of the CM, an increase of 21% in collagen content was determined. To gain more insight in which type of collagen was produced, we stained for collagen I, II, and X (figure 3(A)). Collagen II was only limitedly deposited in the construct with coupled peptide in BM, but was absent in the other conditions (figures S15 and S16). In the samples with collagen II also collagen I deposition was visible, next to the constructs cultured in CM. In BM, collagen I appeared less fibrous and might be present in its procollagen intermediate. Collagen X was expressed in all constructs with no appreciable differences between them. In addition, we observed collagen X turnover, as an enzyme-linked immuno sorbent assay displayed values ranging from 24 to 64 pg collagen X/*μ*g DNA (figure S20(A)).

**Figure 4:**
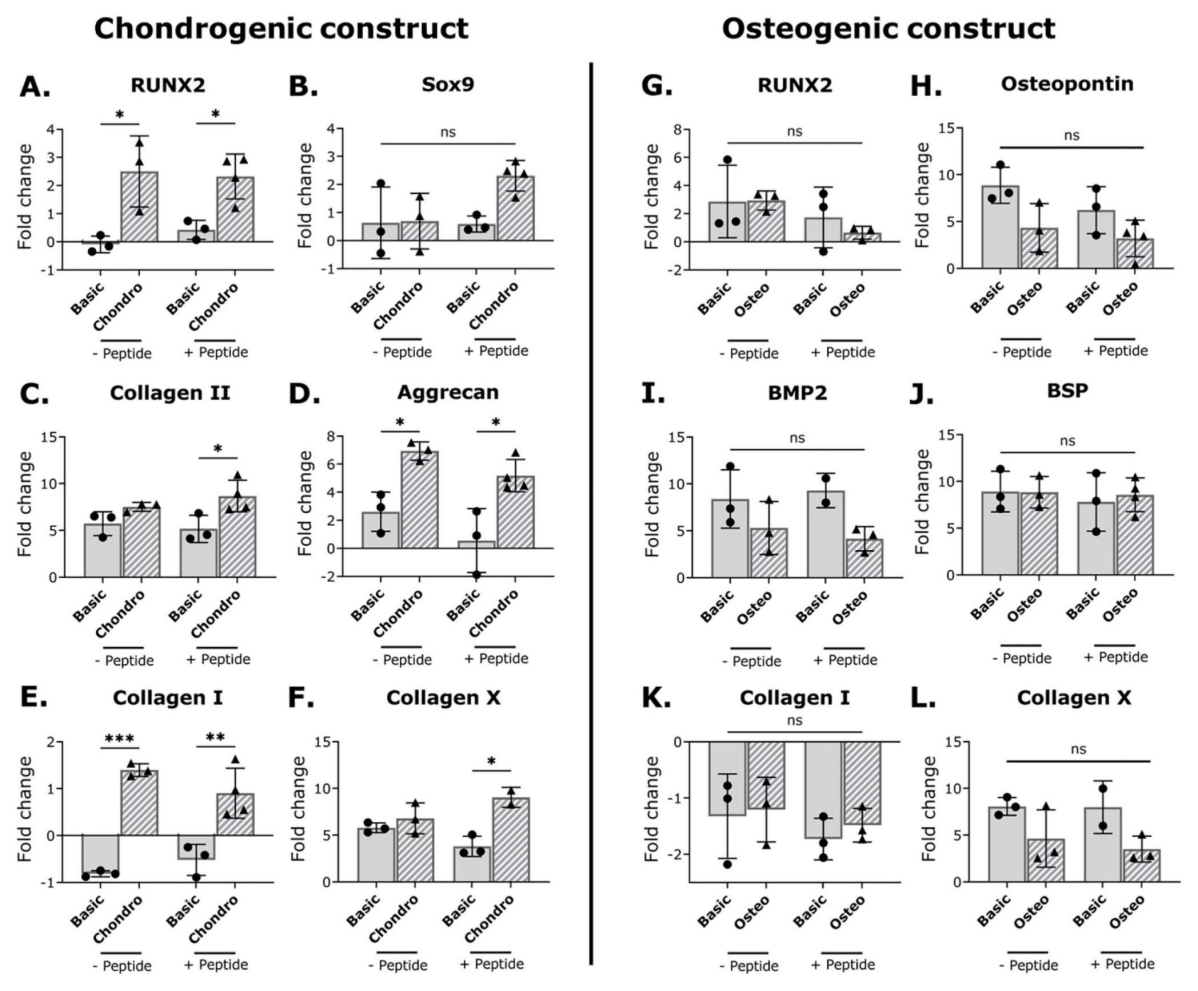
Fold change in relative gene expression levels in the PCLA and PCLM scaffold cell constructs after 21 days. The fold change was determined relative to monolayer cell cultures. In (A)–(F), fold change of chondrogenic markers in the chondrogenic constructs. In (G)–(L), fold change of osteogenic markers in osteogenic constructs, *N ≥* 2. Data is presented as mean *±* SD, ns = non-significant, *p > 0.05, **p > 0.01, **** *p* > 0.0001.

The gene expression profile of cells cultured in chondrogenic constructs was also determined after 21 days (figures 4(A)–(F)). We investigated both the expression of transcription factors considered as early markers as well as the expression of ECM proteins characteristic of chondrogenesis and osteogenic environments. Except for Sox9, we observed a significant effect of the CM on all chondrogenic (Aggrecan and collagen II) and hypertrophy-related (RunX2, collagen I, and X) genes. When assessing the effect of peptide addition in the different media, we only noted a downregulation of Aggrecan in BM, whereas the other genes did not display differences. To assess synergistic effects of the peptide, we determined the (enhanced) ratio as result of differentiation media without and with coupled peptide. We found that Aggrecan and Sox9 had significantly higher ratios when the peptide and CM were combined (figure S21(A)).

Chondrogenesis is induced by upregulation of the Sox9 gene, leading to production of the characteristic collagen II and GAGs [53]. Upregulation of RunX2 induces chondrocyte hypertrophy, and simultaneously assists transformation towards an osteogenic phenotype [54]. Hypertrophic chondrocytes are found in the deep zone with characteristic deposition of collagen X and less collagen II [55]. After 7 days of culture, we stained for both RunX2 and Sox9 transcription factors. In BM, we observed pre-dominantly RunX2 expression, but in the CM also Sox9 was co-expressed albeit that the RunX2 signal was clearly stronger (figure S22). After 21 days, the gene expression of RunX2 and Sox9 had the same pattern with pre-dominantly RunX2 expression instead of Sox9. Moreover, we observed in the CM co-expression of chondrogenic (collagen II and Aggrecan) and hypertrophic genes (collagen I and X). These observations suggested that both chondrogenic and hypertrophic pathways were activated. The ECM deposited when cells were cultured in CM contained a significantly higher amount of GAGs, but the values were lower than 10 *μ*g GAG/*μ*g DNA as reported in other studies [12, 19]. We believe that the anisotropic cell distribution and low cell density resulted in this lower GAG deposition as the dextran-supplemented approach resulted in an increased GAG deposition (figure S23). All conditions displayed collagen X deposition with low remodeling rates, as indicated by the amount in the medium. These observations suggest that the hMSCs are evolving towards a hypertrophic chondrogenic state.

We hypothesized that the evolution towards a hypertrophic phenotype was related to the mechanical properties of the substrate. The PCLA substrates had an approximate stiffness of 88 MPa, which is in the regime of a mineralized OC region [8] and cancellous bone [56], whereas articular cartilage has a compressive modulus of 0.3–0.8 MPa [57]. HMSCs cultured on substrates with a stiffness lower than 10 MPa tend to upregulate chondrogenic markers, while a higher stiffness increases typically osteogenic and therefore also hypertrophic chondrocyte markers [58]. For example, Camarero-Espinosa *et al* indeed demonstrated that a highly resilient poly(ester urethane) AM scaffold, with a compressive modulus of approximately 4 MPa, had an intrinsic chondrogenic effect on hMSCs [59]. In addition, the hMSCs adopted a spread morphology in the scaffolds (figure 3), which is usually associated with induction of an osteogenic pathways of hMSCs such as higher RunX2 activity [60]. The higher RunX2 activity might drive the cells towards a hypertrophic state in an environment with chondrogenic differentiation factors. When hMSCs maintain a rounded morphology, the chondrogenesis process is triggered as Sox9 activity is maintained [61, 62]. The pellets indeed confirmed that after 21 days Sox9 activity was upregulated, yet no RunX2 (figure S24). Moreover, collagen II deposition was clearly visible in the crosssection of a pellet. (figure S25(A)). In the constructs with spread hMSC morphology, collagen II was only slightly observed in constructs with coupled peptide in BM.

Zooming into the intrinsic effect of the peptide, we observed moderate expression of collagen I in the BM, while ASAP was absent. Simultaneously, GAG and collagen deposition displayed increasing trends in BM with coupled peptide. We also compared the differentiation in the constructs to pellet cultures in BM and CM, which is the golden standard for chondrogenesis. The pellets in CM deposited significantly more GAGs and collagen compared to the BM (1.28 ± 0.05 *μ*g GAGs/*μ*g DNA and 3.2 × 10^3^ ± 0.9 × 10^3^ ng collagen/*μ*g DNA, respectively) (figures S25(B) and (C)). The GAG and collagen deposition approached the values of the chondrogenic constructs cultured in BM without peptide, implying a chondrogenic effect on the cells by the material. Notably, the GAG and collagen deposition in constructs bearing peptide and cultured in BM was clearly enhanced to the pellets cultured in CM (~60% and ~50% higher, respectively). Although significant trends were absent in the construct as result of coupled peptide, the results, relatively to the pellets, indicated that the peptide might positively affect chondrogenesis on top of the material.

To enhance the effect of the peptide in future research, we could aim to improve the binding efficiency of TGF-*β* to the sequence. Recently, it was demonstrated that deamination of the chondropeptide, by substitution of the N with a D residue, led to prolonged expression of TGF-*β* [36]. Moreover, this group also described a concentration-dependent effect on differentiation in relation to the amount of binding peptide [35]. The cell density in the pores may also affect the efficiency of the peptide. For example, it was already demonstrated that pore size and cell density were related to differentiation fate of hMSCs [63]. The dextran-supplemented seeding method led to a higher expression of GAGs, indicating an effect of cell distribution. Taken together, the differentiation fate of hMSCs as a consequence of the peptide is likely dependent on additional active parameters, such as the surface concentration or seeding method.

##### 3.3.2.2 Osteogenic constructs

Next, we investigated the expression of osteogenic differentiation markers of hMSCs after incubation of osteogenic constructs in BM or OM. RunX2 is a key regulator in osteoblastic differentiation of hMSCs and bone formation [64]. Characteristic for mature bone formation, compared to hypertrophic chondrocyte differentiation, is high collagen content and mineralization of the matrix. Before hMSCs form mature bone matrix, a gradual transition and maturation of the matrix is observed [65]. To determine the differentiation potential of osteo-peptide and the construct, we assessed hMSC commitment towards osteogenic lineage by investigating characteristic markers from the early to late maturation phases. Simultaneously, collagen X deposition and remodeling were assessed to determine if (hypertrophic) chondrogenic pathways were still active.

After 7 days, we observed a predominant expression of RunX2 compared to Sox9 in all conditions (figure S26). We hypothesized that the cells in these constructs were evolving towards an osteogenic phenotype, especially when culturing in OM. In OM, PCLM without coupled peptide afforded an ALP activity value, a general mineralization marker, of 3.7 × 10^3^ ± 0.3 × 10^3^ and with coupled peptide a value of 3.1 × 10^3^ ± 0.6 × 10^3^ AU/*μ*g DNA. The activity in BM was significantly lower (without peptide: 3.2 × 10^2^ ± 0.4 × 10^2^ vs with peptide: 3.7 × 10^2^ ± 0.2 × 10^2^ AU/*μ*g DNA) (figure 2(C)). However, when we assessed macroscopic mineralization, none was observed in any construct after 21 days (figure S27). We assessed expression of OPN and osteocalcin as mid- and late osteogenic markers. We observed expression of OPN in all conditions after 21 days (figure S28). OCN was only expressed in BM, but 2D controls also showed the presence of OCN in BM, suggesting that the expression could be related to the cell density (figure S29). Finally, we determined the type of collagen being deposited. We observed a higher collagen I deposition in the OM. Collagen X seemed present in all conditions, but it was less abundantly in the OM (figure 3(B)). The higher deposition of collagen I and lower deposition of collagen X, and vice versa, likely explains the absence of differences in total collagen deposition (figure 2(E)). The turnover of collagen X was only significantly enhanced in OM (without peptide: 1.6 × 10^2^ ± 0.6 × 10^2^ and with peptide: 1.1 × 10^2^ ± 0.4 × 10^2^ pg/*μ*g DNA) compared to BM (without peptide: 2.6 × 10^1^ ± 0.1 × 10^1^ and with peptide: 2.7 × 10^1^ ± 0.1 × 10^1^ pg/*μ*g DNA) (figure S20(B)).

The gene expression in the osteogenic constructs was further investigated after 21 days. We investigated both the expression of transcription factors considered as early markers as well as late osteogenic environments. Surprisingly, we observed a down regulation of OPN, BMP2, Sp7, and OCN after 21 days (figures 4(G)–(L) and S30). 2D controls showed no clear significant upregulation of osteogenic genes, except for bone sialoprotein (BSP) (figure S31). Unlike the chondrogenic genes, no synergistic effects were observed in the osteogenic genes (figure S21(B)).

The OM was the dominant factor in driving hMSCs differentiation in osteogenic constructs. The enhanced activity of ALP, presence of OPN, and deposition of collagen I indicated that the ECM was undergoing a mineralization process in OM. Moreover, collagen X was less abundantly present in OM, compared to the chondrogenic constructs, indicating a high turnover rate. Collagen X is an intermediate collagen type during the endochondral ossification, the transition from calcified cartilage towards new bone [66]. The lower deposition and higher turnover indicated that the hMSCs are progressing towards osteogenesis in OM. In BM, the collagen X presence and low ALP activity indicated that the cells adopted a hypertrophic chondrogeniclike phenotype. Although there was OPN present in this medium, we attributed it to its involvement also in proliferation of hMSCs [67]. The constructs with coupled peptide did not display differences compared to the constructs without peptide.

We believed that additional parameters such as cell distribution, peptide sequence, or surface density might be involved in dysfunctioning of this peptide. In a preliminary experiment, we initially assessed the effect of cellular distribution via the dextransupplemented seeding method. The ALP activity displayed the same pattern, yet no peptide effect was observed (figure S32). Our results implied that, unlike chondrogenesis as seen by the GAG deposition, osteogenesis was not affected by the seeding method [39]. Secondly, we here selected the ‘wrist’ epitope peptide of the BMP2 protein to induce osteogenic differentiation. Some conflicting literature was published regarding this peptide. Lee *et al* demonstrated that the wrist epitope bound to the receptor and led to downstream Smad phosphorylation [37], but Madl *et al* claimed dysfunctioning of the peptide as no effect on ALP activity was observed when immobilized in their hydrogel [68]. Although the latter study also incorporated an RGD epitope, a recent study demonstrated that the wrist epitope is only effective when it is co-presented at a surface density of 38 pmol cm^−2^ with the RGD in close proximity [69]. Overall, the intrinsic effect of the osteo-peptide to induce osteogenic differentiation seemed questionable. Besides the wrist epitope, the BMP2 protein also contains a ‘knuckle’ epitope. Studies did report successful osteogenic differentiation of hMSCs in response to this epitope [70, 71]. The knuckle epitope was also anchored on AM fibers and at a surface density of 75 pmol cm^−2^ a positive trend was displayed in the ALP activity [49]. Notably, it again indicated that the surface density of the peptide may affect the differentiation. Taken together, the peptide sequence and surface require further investigation to actively induce osteogenic differentiation in AM constructs.

### 3.4 Biocompatibility *in-vivo*

In order to test the validity of the constructs to be exploited *in vivo*, we tested the biocompatibility subcutaneously in a rat model. Bare scaffolds with or without peptide were implanted to assess the foreign body response to the materials, cellular infiltration, and matrix deposition. In addition, we implanted scaffolds with pre-seeded hMSCs to compare the deposited matrix of the infiltrated and pre-seeded cells. The scaffolds were fabricated in a discrete fashion having each side of the scaffold a different chemistry to be able to detect a clear response to the two different polymers and chemistries (figure S33).

**Figure 5:**
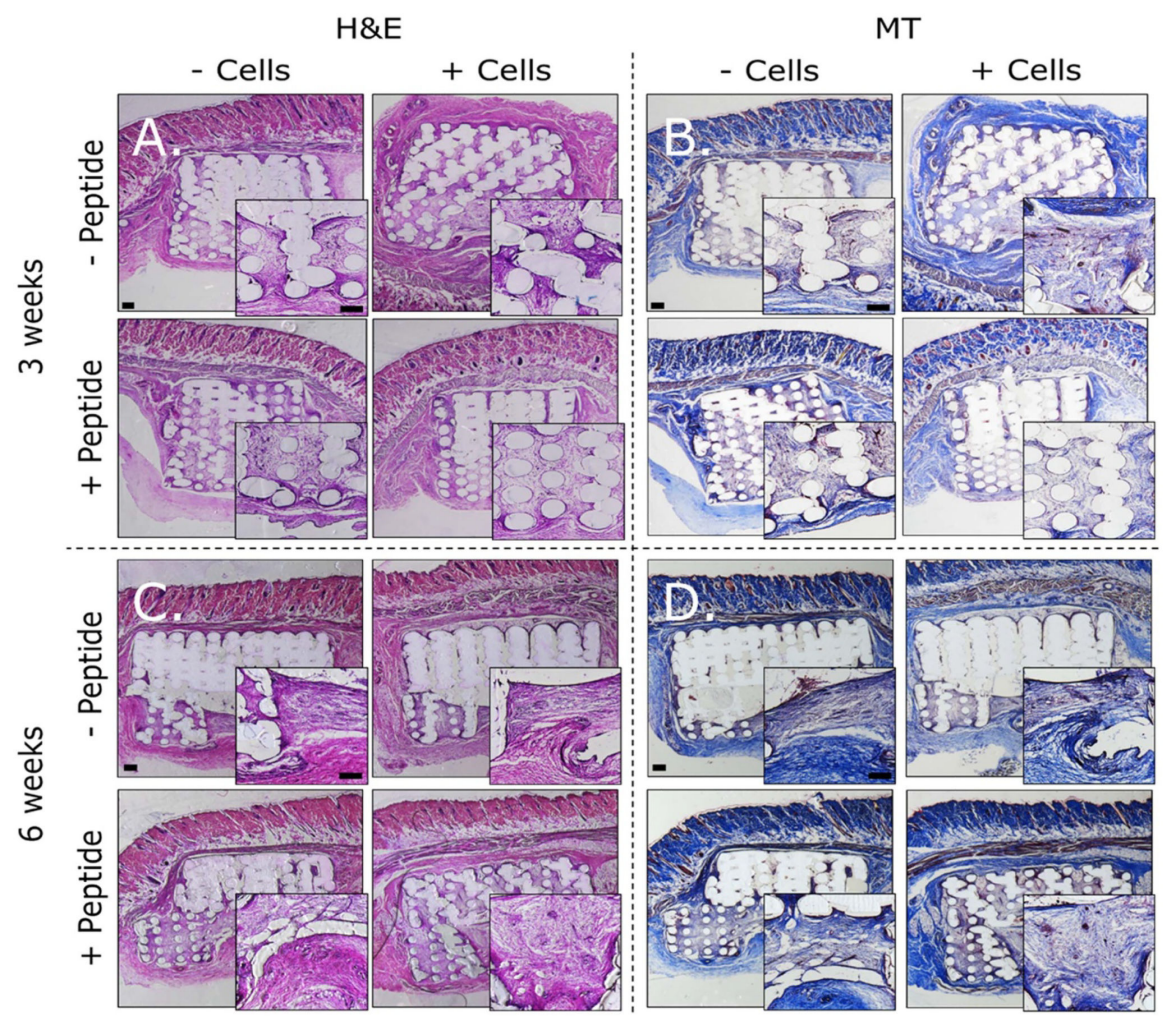
Biocompatibility study of PCLA/PCLM discrete gradient scaffolds in a subcutaneous rat model up to 6 weeks implantation. Sections were stained with either H&E (left) or MT (right). The top part of the scaffold is PCLA and the bottom part, with hole to mimic the bone marrow cavity, is PCLM. Scaffolds without and with peptide (A) and (C), and without or with pre-seeded hMSCs (B) and (D) were used. Scale bar = 300 *μ*m. *N* = 8.

Overall, we observed low presence of inflammatory cells around the fibers. In addition, the fibrous capsule around the full constructs remained limited after 3 and 6 weeks, especially in the constructs bearing peptide (figure 5). Over the course of 6 weeks, we observed high tissue infiltration and newly deposited collagen behind the infiltrated frontline in the constructs without pre-seeded cells (figures 5(C) and (D)). However, the infiltration was higher in the osteogenic compared to the chondrogenic component, especially in the constructs bearing peptide. In the constructs with pre-seeded cells, the constructs were more populated with cells, but we did not observe difference in the type of deposited tissue based on the MT staining compared to the empty implanted scaffolds. In the osteogenic components, we did observe initial vessel formation, as evidenced by the presence of erythrocytes, independently of the presence of the peptide. In the PCLA component, we observed thicker collagen deposition around the fiber in the constructs bearing peptide, potentially implying a positive effect of the peptide. The constructs bearing peptide displayed a thicker collagen deposition around the fibers, independently on presence of pre-seeded cells. Taken together, the polymer and peptide had good biocompatibility with low foreign body responses, making them safe to explore further *in-vivo*. Additionally, the constructs had good (lateral and medial) porosity to allow for tissue integration as well as new ECM deposition.

### 3.5 Printing continuous material gradients

**Figure 6:**
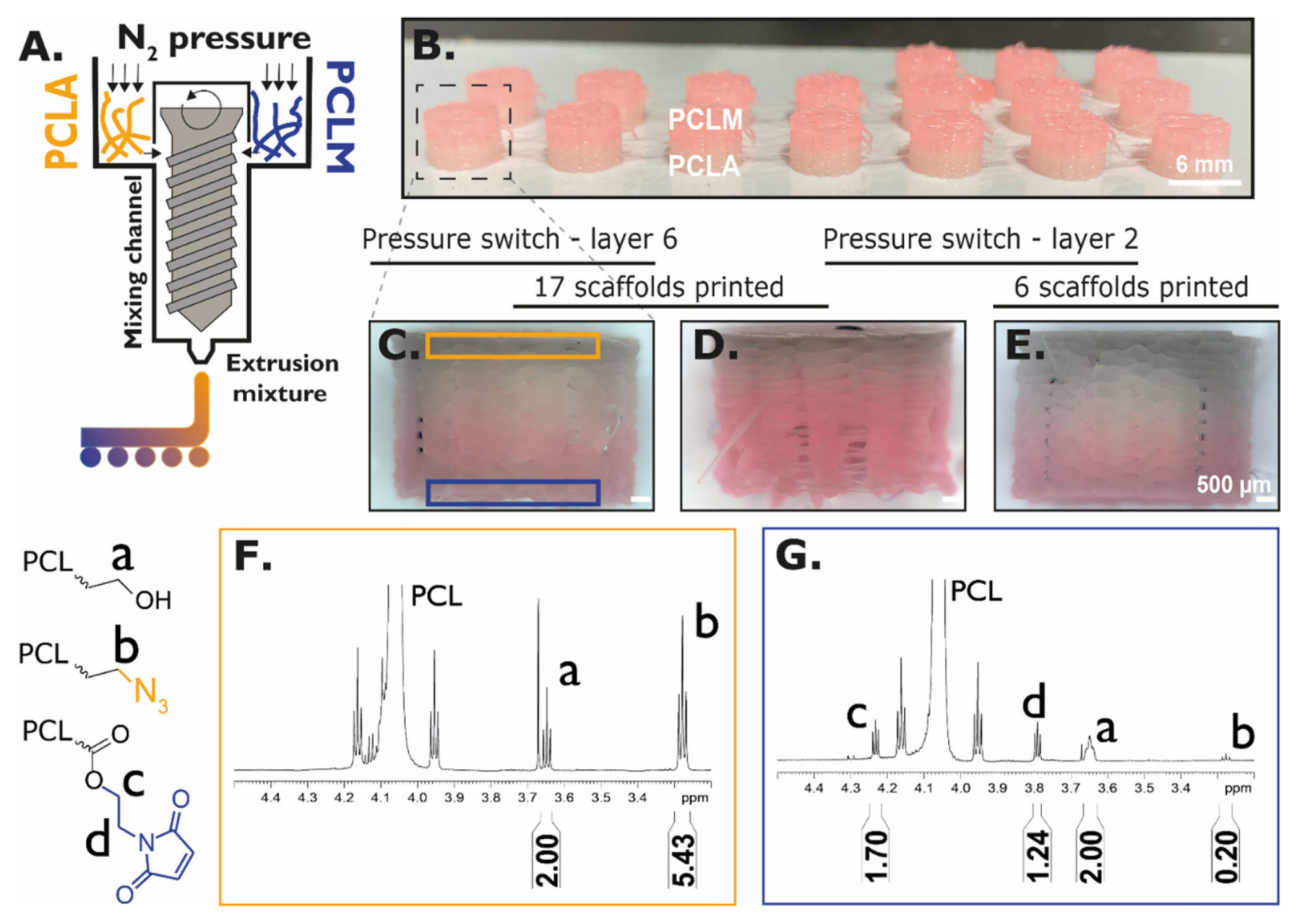
Fabrication and characterization of scaffolds containing continuous material gradients. (A) Schematic of the in-house developed printer-head. Two reservoirs with individual pressure control accommodating a PCLA or PCLM melt. Varying the pressure between the reservoirs enables real time changes in the extruded polymer mixture. (B) Photograph of medium throughput fabricated ready-to-use scaffolds. (C)–(E) Using a dispersed dye in the PCLM melt, the presence of material gradients is visualized. The timing of the pressure switch or the number of printed scaffolds determined the gradient distribution. (F) and (G) The ^1^H NMR spectra of a top (yellow box) and bottom (blue box) layer of a gradient scaffold as shown in (C). The ‘b’ protons in (G) indicate that PCLA was replaced for about 95% by PCLM.

Ultimately, we aim to fabricate a single OC scaffold that guides hMSCs towards both chondrogenic and osteogenic phenotypes on opposite sides in response to peptides. However, simply merging the two scaffolds, as done in the *in-vivo* study, would create a discrete material and peptide gradient. We hypothesized that a gradual transition from chondrogenesis towards osteogenesis is preferred to create a more biomimetic construct. To this end, we aimed to create a continuous peptide (biochemical) gradient across a scaffold. We used an in-house developed printer-head with two reservoirs to create AM 3D scaffolds having a continuous material gradient of PCLA and PCLM (figure 6(A)). Due to the presence of click type groups on the surface, we could exploit these in creating biochemical gradients for OC applications.

In a single run up to 17 cylindrically shaped scaffolds were manufactured (figure 6(B)). In this process, the timing of the pressure switch and the number of printed scaffolds in a single run determined the location as well as distribution of the gradient along the *y*-axis of the scaffolds. Addition of a dye in the PCLM melt was used to show the gradient introduced in the scaffolds (figures 6(C)–(E)).

Scaffold gradients of PCLA and PCLM were confirmed by NMR analysis of samples of opposing sides. The PCLM side showed that the PCLA content was reduced by 95% (figures 6(F) and (G)). Using the images depicted in figures 6(C)–(E), gray intensity profiles were used to determine the location of the material gradient in the scaffolds. After an initial delay following the pressure switch the signal intensity continuously decreased until a plateau was reached (figure S34). When printing 17 scaffolds in a single run, the gradient comprised a height of approximately 600 *μ*m, which approximately corresponded to three layers. The timing of the pressure switch only affected the location within the scaffold, but not the gradient length. When we fabricated less scaffolds simultaneously, we observed a broader distribution of the gradient (1400 *μ*m) along the scaffold.

The extruded volume for a full material transition could be roughly determined by the length of gradient including the delay after the pressure switch signal (figure S34). Based on the g-code design, the layer height of the scaffolds was 200 *μ*m and the extruded volume of one scaffold layer was 0.0028 cm^3^. When printing 6 or 17 scaffolds, the delay and gradient height corresponds to roughly 13 (2600 *μ*m) or 7 (1400 *μ*m) printed layers in one scaffold, respectively. To obtain the total amount of extruded volume to switch material, we multiply the layers by the total manufactured scaffold in the full run. Thus, the total deposited volume of material for a full transition corresponds to 0.33 and 0.22 cm^3^, respectively. By using the volume of the extrusion chamber, corresponding to 0.16 cm^3^, we found that 1.4–2.1 times of this volume could be used to predict the switch volume (figure S35). A value above 1.0 was expected, since first the remaining PCLA had to be extruded before the PCLM slowly appeared. Taken together, we concluded that we created continuous material gradients in a controlled fashion with this AM platform.

### 3.6 Mechanical characterization of gradient scaffolds

**Figure 7:**
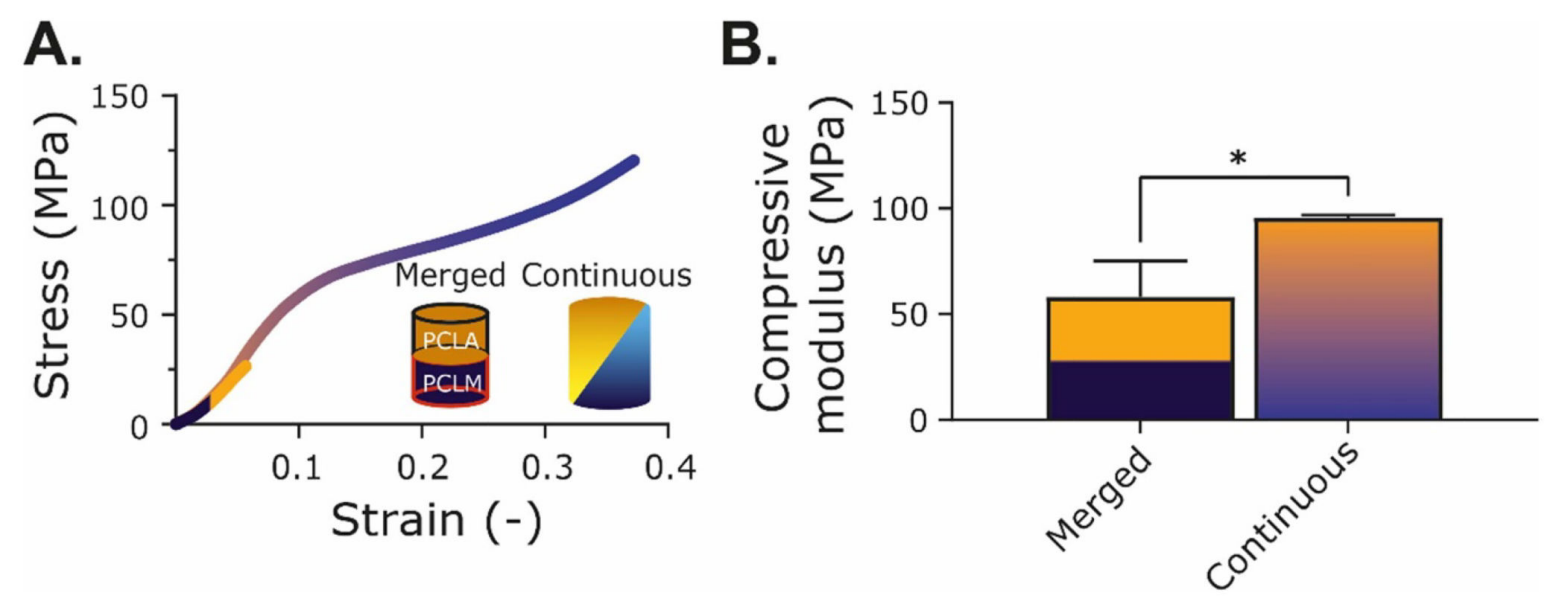
Mechanical analysis of scaffolds using compressive testing. (A) Representative stress–strain curves. All curves can be found in the supporting information (figures S6(C) and (D)). (B) The average compressive modulus of each group, *N* = 3. Data is presented as mean *±* SD, *p < 0.05, **p < 0.01.

Discrete material gradients in AM scaffolds are fabricated by consecutive printing using multiple print heads [19]. To strengthen cell–biomaterial interfaces biocompatible glues are sometimes used [72, 73], but when multiple layers are combined this process becomes unpractical. Manufacturing these discrete multi-layered scaffolds creates weak interfaces, which are also prone to delamination due to mismatching mechanical properties. In a compressive test, we compared the mechanical behavior of different gradient types (discrete and continue) in cylindrically shaped scaffold (figure 7(A)). The fiber dimensions of continuous gradient scaffolds were optimized to resemble the PCLA and PCLM scaffolds closely (table S2).

Representative stress–strain curves are presented in figure 7(A). We observed a compressive modulus of 58 ± 17 MPa for the PCLA-PCLM merged scaffold and 96 ± 1.1 MPa for the gradient scaffold (figure 7(B)). After an initial elastic response, the gradient scaffold displayed a pore collapse followed by densification (video S1). The stress strain curve was similar to the PCLM scaffold (figure 1(B)). The merged scaffolds started to disintegrate on the PCLA side (video S2), as also observed with neat PCLA scaffolds, leading to a strain at break of 0.056 ± 0.017. The adoption of the lowest stiffness value of multimaterial scaffolds comprising discrete gradients was also reported in other studies [19, 74]. These results implied that the gradient scaffolds were stiffer with higher strain at break. Notably, both scaffolds contained about a 1:1 w/w ratio of PCLA to PCLM, whereas the brittleness was only lost in the gradient scaffold. Furthermore, the gradient scaffolds did not show delamination, which was an issue during preparation of the merged scaffolds. These results implied that gradual mixing of different thermoplastic polymers with our AM technology might lead scaffolds with higher integrity.

## 4 Conclusions

In this study, we successfully installed clickable groups on the surface of AM PCLA and PCLM scaffolds for OC applications. We attached fluorescent dyes on the surface finding surface density in the 10^2^ pmol cm^−2^ regime. Thereafter, we switched the dyes for differentiation-inducing peptides to induce chondrogenesis (PCLA scaffolds) and osteogenesis (PCLM scaffolds) in hMSCs. In the hMSC-PCLA constructs in BM and CM, we found that cells are driven towards a hypertrophic chondrogenic phenotype, as characterized by the collagen X deposition, which we ascribed to the substrate stiffness of the scaffolds. Besides in CM, we observed collagen I deposition in the scaffolds with coupled peptide in BM. In this condition, we also observed some collagen II expression, which was absent in all other constructs. Additionally, the total collagen and GAG deposition displayed an increasing trend in BM with coupled peptide. Furthermore, the collagen and GAG deposition was also enhanced relatively to the chondro pellets in CM. Thus, we concluded that the chondro-peptide had a limited intrinsic effect on the hMSC fate. In the hMSC-PCLM constructs, we only observed an effect of the differentiation media and no intrinsic effect of the osteo-peptide on osteogenic differentiation. Besides *in-vitro* differentiation studies, *in-vivo* studies prooved good biocompatibility of the material and peptides, including good porosity in terms of cellular integration. Taken together, we concluded that further investigation of active parameters, such as substrate stiffness as well as effects of surface concentration of our peptides on cells is required.

Ultimately, we aim to fabricate a scaffold containing a (biochemical) gradient of two bioactive peptides to gradually guide hMSC differentiation from a chondrogenic towards an osteogenic pathway. Although the *in-vitro* differentiation results require further research to evoke significant effects on cells, we manufactured continuous PCLA-PCLM gradient via a novel extrusion-based AM technology, with control over the gradient distribution. Consequently, we simultaneously generated a gradient of the addressable group across the scaffold. These groups could in future studies be used to generate biochemical gradient to guide cell differentiation across a scaffold. Despite initially aiming for a full OC constructs, we believe that, based on the *in-vitro* differentiation results, these scaffolds are potentially more suited to mimic the area around the tidemark. Importantly, due to the versatility of click chemistry, we created a chemical system that is amenable to other bioactive molecules, which makes these scaffolds also interesting for other tissues containing gradients. Another advantage of the gradual material transition is the absence of interfaces in these scaffolds, making them more resistant to delamination. A discrete PCLA-PCLM gradient did show signs of delamination.

Moreover, continuously produced PCLA-PCLM scaffolds displayed higher mechanical properties compared to the discrete gradient scaffolds. In future studies, it would be interesting to also investigate the mechanical behavior when mixing two more fundamentally different polymers to approach the mechanical properties of the OC unit more closely.

## Supplementary Material

Sch S1, Tab S1-S4 and Fig S1-S35

## Data Availability

The data that support the findings of this study are openly available in DataVerse at https://doi.org/10.34894/YISKVU, reference number [75].
