# Supplementary material for: Installation of click-type functional groups enable the creation of an additive manufactured construct for the osteochondral interface
Source: Biofabrication. Author manuscript; Available in PMC 2026 Jun 27. (PMC7619215; doi:10.1088/1758-5090/aca3d4)
Supplement: Sch S1, Tab S1-S4 and Fig S1-S35 [file EMS214257-supplement-Sch_S1__Tab_S1_S4_and_Fig_S1_S35.pdf]

Supporting information

Installation of click-type functional groups enable the creation of an additive manufactured construct for the osteochondral interface

Ivo A.O. Beeren[1], Pieter J. Dijkstra[1], Ana Filipa H. Lourenço[1], Ravi Sinha[1], David B. Gomes[1], Hong Liu[1,2], Nicole Bouvy[2], Matthew B. Baker[1], Sandra Camarero-Espinosa[1, 3, 4], Lorenzo Moroni[1*]

[1]*Department of Complex Tissue Regeneration, MERLN Institute for Technology-Inspired Regenerative Medicine, Maastricht University, 6229 ER Maastricht, The Netherlands.*

[2]*Department of General Surgery, Maastricht University Medical Center, 6200 ND Maastricht, The Netherlands.*

[3]*POLYMAT, University of the Basque Country UPV/EHU, Avenida Tolosa 72, Donostia / San Sebastián 20018, Gipuzkoa, Spain.*

[4]*IKERBASQUE, Basque Foundation for Science, Bilbao 48009, Spain*

*Corresponding author address: l.moroni@maastrichtuniversity.nl

Table of Contents

1. Methods 3

1.1 Coupling of dyes to polymers 3

1.2 Scaffold fabrication for in-vivo studies 3

1.3 Dextran-supplemented seeding method & biological evaluation 3

1.4 Chondro pellet culture 4

1.5 Biological evaluation of chondro pellets 4

1.6 Biological evaluation in-vitro constructs 4

1.6.1 Alizarin red S staining (ARS) 4

1.6.2 Safranin-O staining 5

1.6.3 Collagen X content in media 5

2. Polymer synthesis and characterization 6

3. Scaffold characterization 10

4. In-vitro hMSC study 14

4.1 Cell adhesion and proliferation 14

4.2 Cell differentiation 16

5. Tables 32

1. Methods

1.1 Coupling of dyes to polymers

PCL-Azide (PCLA, 27 mg, 1.17x10^{-3} mmol) was dissolved in 1 mL of DMF. Consecutively, Cu(II)$SO_4 \cdot 5H_2O$ (1.61 mg, 6.43x10^{-3} mmol), sodium ascorbate (12.7 mg, 6.43x10^{-2} mmol), and alkynated MegaStokes dye 673 (3.22x10^{-3} mmol, from a 10 mg.mL^{-1} dimethyl sulfoxide (DMSO) stock solution) were added to the solution and the mixture was stirred overnight at RT. The product was precipitated in water, re-dissolved in DMF, precipitated again in water, and the precipitate was extensively washed with water until the solution was colorless. Finally, the remaining water was removed by freeze-drying.

PCL-Maleimide (PCLM, 150 mg, 4.87x10^{-3} mmol) was dissolved in 5 ml of DMF. Then, 1% v/v triethylamine (TEA, VWR), 0.25% w/v tris(2-carboxyethyl) phosphine hydrochloride (TCEP), and 7-mercapto-4-methylcoumarin (4.65 mg, 2.42x10^{-2} mmol, from a 10 mg.mL^{-1} DMSO stock solution) were added. The mixture was stirred at RT overnight and the product was precipitated in an excess of methanol. The precipitated polymer was washed two times with methanol, three times with DMSO, and then two times with water. Finally, the remaining water was removed by freeze-drying.

Size exclusion chromatography in combination with a photodiode array detector was used to confirm successful coupling of the dyes on the polymer. We used a GPC Shimadzu system comprised of an autosampler, a Shodex KD-G 4A guard column (4.6 x 10 mm) with 8 μm beads, followed by two Shodex KD-802 (5 μm, 8 x 300 mm) and KD-804 (7 μm, 8 x 300 mm) columns, a refractive index detector, and a photodiode array detector at 50 °C. DMF containing 0.1 wt% LiBr was used as eluent at a flow rate of 0.5 mg.mL^{-1} at 50 °C, and using linear poly(methylmethacrylate) standards as molecular weight standard. During the run, we continuously measured the λ_{exc}= 506 nm and λ_{exc}= 329 nm for PCLA and PCLM, respectively.

1.2 Scaffold fabrication for in-vivo studies

For the in-vivo studies, we printed on a Bioscaffolder (sysENG, Germany) a PCLA and PCLM grid using the same parameters as described in section 2.3.1. PCLA was extruded through a 25G nozzle at 86 °C using a pressure of 4.1 bar, a translation speed of 400 mm.min^{-1}, and a auger screw rotation of 80 RPM. PCLM was extruded through a 25G nozzle at 82.5 °C using a pressure of 4.3 bar, a translation speed of 425 mm.min^{-1}, and an auger screw rotation of 80 RPM. Disks (6 mm in diameter and 2 mm in height) were punched from the grid. The PCLM scaffolds contained a central 2 mm inner hole (see figure S33) to mimic the in vivo bone marrow cavity.

1.3 Dextran-supplemented seeding method & biological evaluation

Scaffolds, PCLA and PCLM, were sterilized in 70% ethanol overnight and washed with PBS. Part of the scaffolds were sterile functionalized with peptides as described in the section 2.4.2 of the main text. The scaffolds were dried on sterilized filter paper. The scaffolds were placed in non-treated hydrophobic tissue culture well-plates. A cell suspension was prepared in dextran-supplemented media (= basic medium (BM) containing 10% w/v Dextran (500 kDa, Pharmacosmos)). We seeded as a drop of 150.000 cells on top of each scaffold. The volume of

the seeding droplet approached the total scaffold pore volume. Cells were left to attach for 4 h in an incubator at 37 °C and 5% CO_2. Thereafter, 1.5 ml of media (BM + 200 µm ASAP) was added to the wells with scaffolds. The next day, the scaffolds were placed in fresh wells plates. We cultured PCLA scaffolds in CM and PCLM in BM and OM, as described in 2.5.3 of the main text.

After 24h and 7 days, we performed a DNA assay according to 2.5.4.2 on all these constructs. After 7 days, we assessed cell distribution via an AlexaFluor Phalloidin (1:100, Phal-488) staining according to the protocol described in 2.5.6 of the main text. After 21 days, PCLA scaffolds in CM were evaluated by a GAG assay (section 2.5.4.3) & PCLM scaffolds by an ALP assay (section 2.5.4.1).

1.4 Chondro pellet culture

As a 'gold standard' for in vitro chondrogenesis, hMSCs were cultured on pellets as a positive control. To form pellets, 0.25 x 10^6 cells in 0.5 mL BM were placed in a 2 mL Eppendorf tube with a small hole in the lid, created by a needle. The cells were spun down to the bottom of the tube and left overnight in an incubator at 37 °C and 5% CO_2, forming a round pellet. The medium was carefully replaced with either basic or chondrogenic media. The media was refreshed every 2-3 days.

1.5 Biological evaluation of chondro pellets

DNA, GAG and HYP assays were performed according the procedure described in sections 2.5.4.2, 2.5.4.3, and 2.5.4.4 of the main text.

For RT-PCR evaluation, media was removed, 1 mL Trizol was added, and the pellets were stored at -80 °C. Pellets were crushed prior to RNA isolation, according the procedure described in section 2.5.5 of the main text.

For immunofluorescent staining, cell pellets were fixated in 4% PFA in PBS. Samples were initially transferred to a cassette and dehydrated in a series of: 70% ethanol, 95% ethanol, two times in 100% ethanol, and two times in xylene, for 30 min each. Finally, the scaffolds were embedded in paraffin and frozen overnight. The pellets were cut in sections of 7 µm and collected on microscopy slides. Prior to staining, sections were re-hydrated by consecutively submerging the slides for 5 min in two times HistoClear, two times 100% ethanol, 95% ethanol, 70% ethanol, and double distilled water. The same staining procedure, as described in 2.5.7 in the main text, was used for the sections, except that the time for permeabilization and the time of incubation with the secondary antibody were reduced to half. The samples were finally mounted with ProLong® Diamand Antifade mountant to prevent drying out of samples. The samples were imaged under an automated epi-fluorescent Nikon Eclipse Ti2-E microscope.

1.6 Biological evaluation in-vitro constructs

1.6.1 Alizarin red S staining (ARS)

To visualize calcification pockets, an ARS staining was performed. The media was aspirated from the wells, then the samples were rinsed with PBS, and fixated in 4% v/v PFA for 30 min. After rinsing the samples with water, scaffolds were cut in half and submerged in 60 mM ARS (pH 4.1-4.3) for 20 min. The constructs were washed with water to remove the

excess ARS and left in water. Samples were imaged using a stereomicroscope (Nikon SMZ800 with Q-imaging Retiga 1300 camera).

1.6.2 Safranin-O staining

Chondrogenic constructs were fixated in 4% PFA for 30 min and cut along the y-axis. Samples were stained for 10 min in Weigert's iron hematoxylin solution to counterstain the cell nucleus black, washed thoroughly in running tap water, stained for 3 min with Fast Green solution (1 mg in 1% v/v acetic acid), and quickly dipped in 0.5% acetic acid. Finally, samples were stained for 5 min in a 0.1% v/v solution of safranin-O, after which they were rinsed with PBS until the PBS was clear. Samples were imaged using a stereomicroscope (Nikon SMZ800 with Q-imaging Retiga 1300 camera).

1.6.3 Collagen X content in media

Collagen X content in the media was determined using ELISA kits (Novus Biologicals), according the manufacturer's protocol. Media was collected after 21 days of cell culturing in the constructs, spun down at 1000 rcf, and the supernatant was stored at -80 °C until analysis.

2. Polymer synthesis and characterization

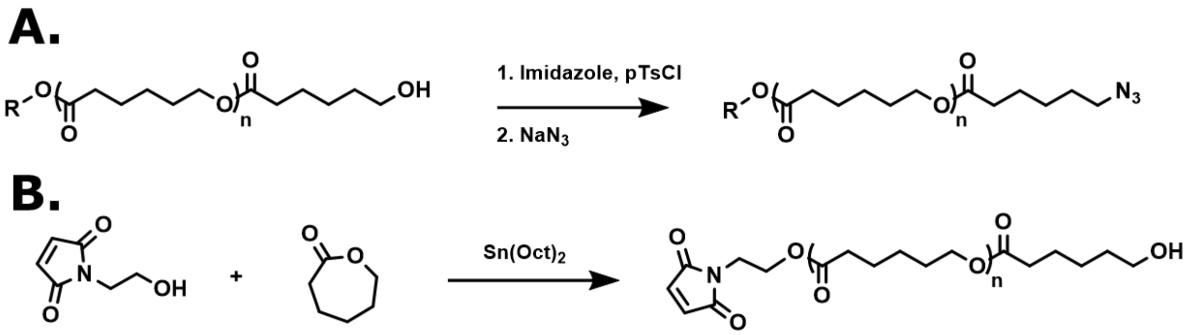

Scheme S1. Reaction schemes: (A) PCL-Azide (PCLA) was synthesized by activation of the hydroxyl group and subsequent reaction with sodium azide. (B) PCL-Maleimide (PCLM) was synthesized by ring opening polymerization of ε-caprolactone using N-(2-hydroxyethyl)maleimide as an initiator and stannous octoate as a catalyst.

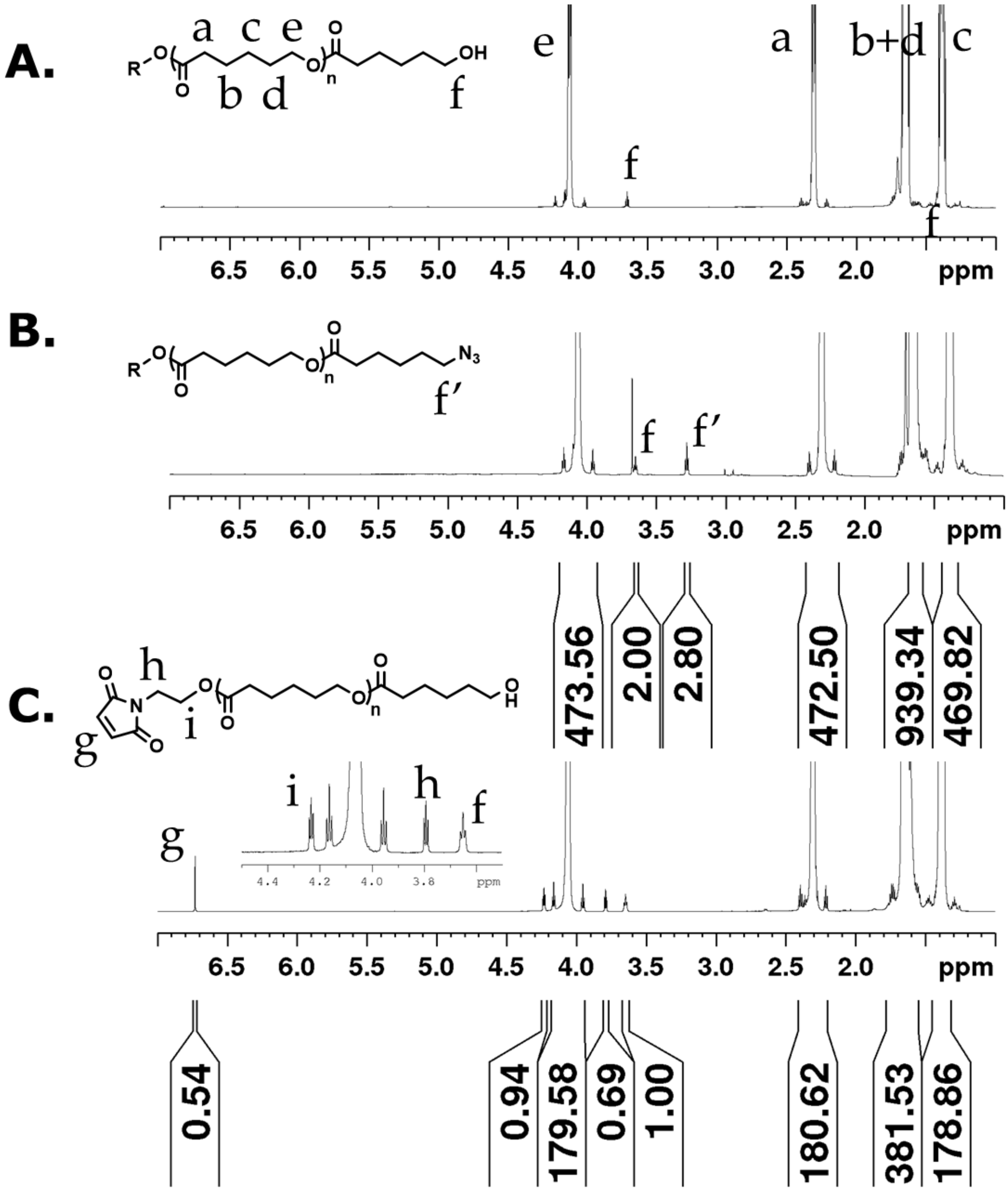

Figure S1. ^{1}H NMR spectra of (A) PCL, (B) PCLA with a degree of substitution of azide groups of 65%, and (C) PCLM with a degree of substitution of maleimide groups of 70% ($CDCl_3$).

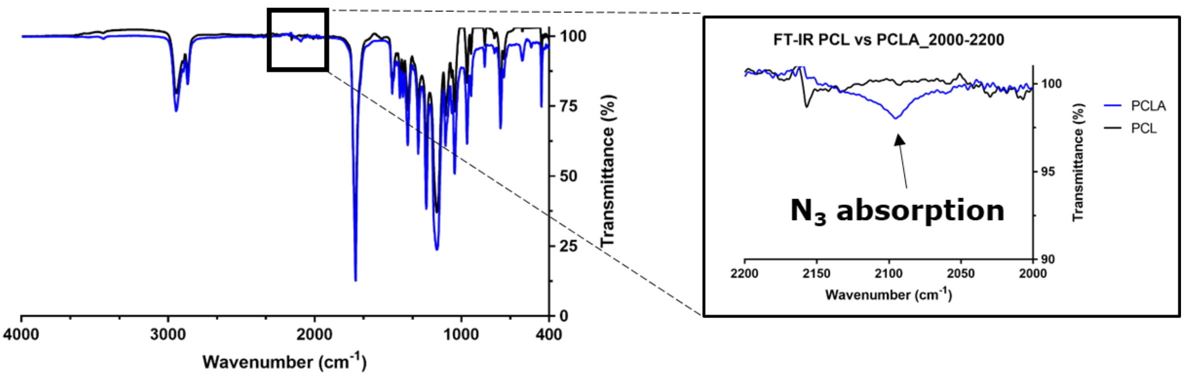

Figure S2. FT-IR spectra of PCL and PCLA.

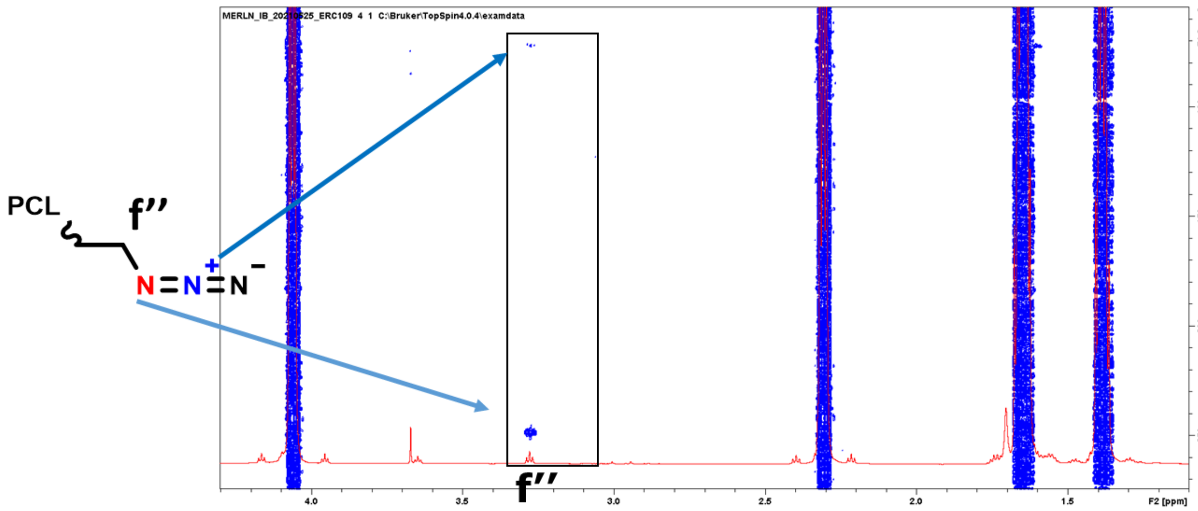

Figure S3. ^{1}H-^{15}N HMBC spectrum of PCLA ($CDCl_3$). The nitrogen shifts correspond closely to reported values of an alkyl azide.

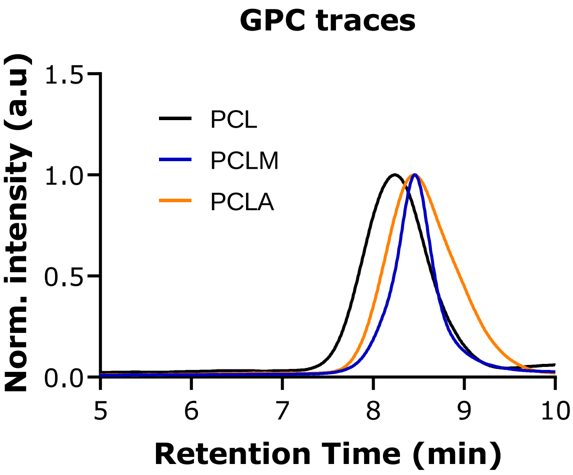

Figure S4. SEC traces of PCL, PCLA, and PCLM.

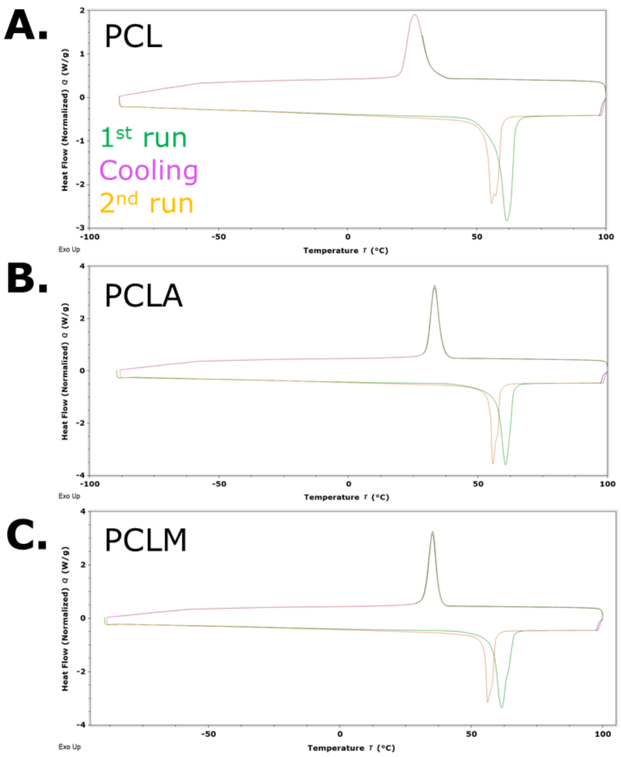

PCL	Peak Temp. (°C)	Enthalpy (J/G)
1st heating run	61.6	93.7
Cooling	26.0	66.5
2nd heating run	55.8	58.6

PCLA	Peak Temp. (°C)	Enthalpy (J/G)
1st heating run	60.7	95.2
Cooling	33.3	70.8
2nd heating run	55.7	67.6

PCLM	Peak Temp. (°C)	Enthalpy (J/G)
1st heating run	61.6	100.7
Cooling	35.3	64.6
2nd heating run	56.3	60.6

Figure S5. DSC heating and cooling thermograms. T_m and T_c values are presented in the tables. (A) PCL, (B) PCLA, and (C) PCLM.

3. Scaffold characterization

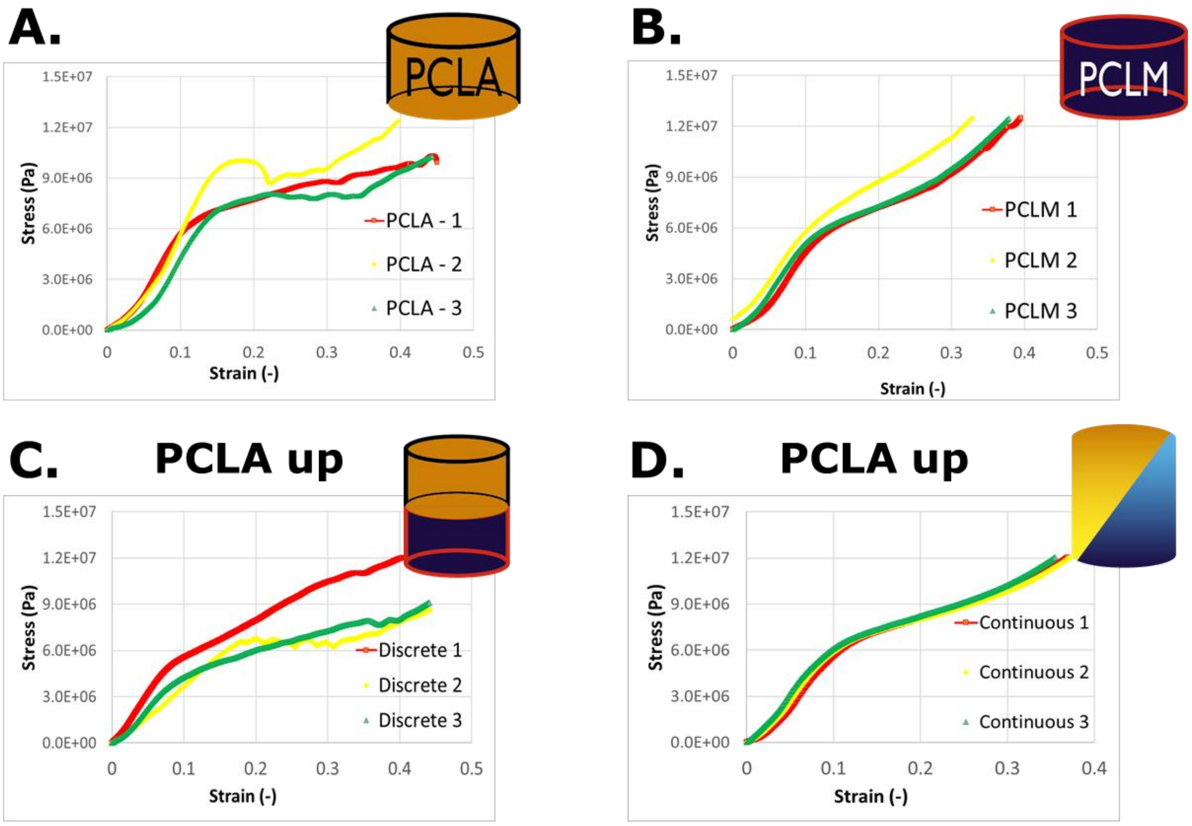

Figure S6. Compression stress-strain curves of (A) PCLA, (B) PCLM, (C) Discrete gradient, and (D) Continuous gradient scaffolds. The PCLA component was always facing upwards during the test. N=3.

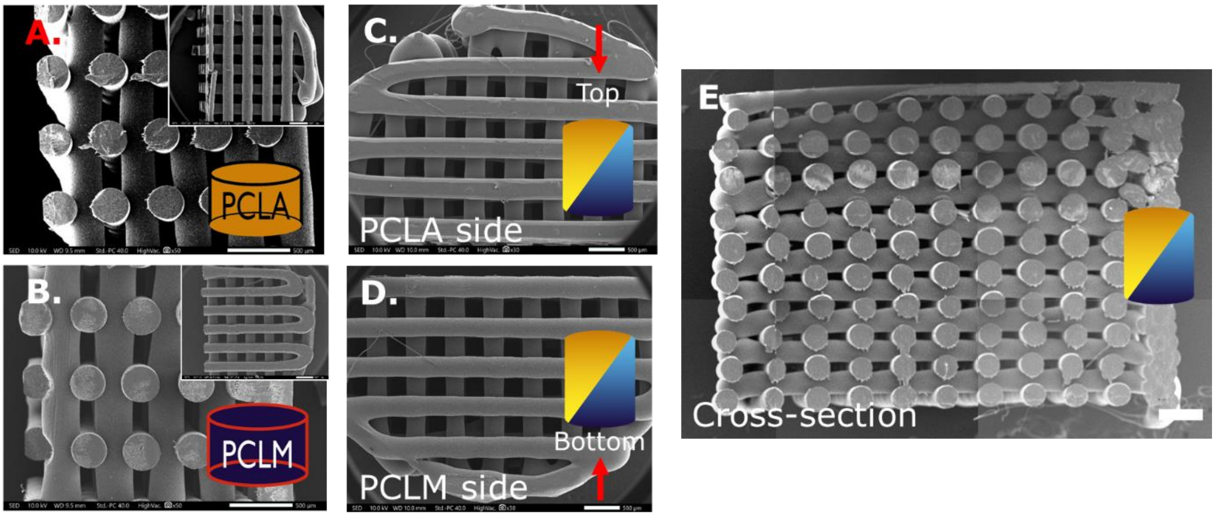

Figure S7. SEM images (A) – (C) of scaffolds having a continuous material gradient of PCLA and PCLM, (D) PCLA, and (E) PCLM scaffolds. (B, C) present the top and bottom view of the continuous gradient scaffold. These images were used to calculate the scaffold dimensions, see table S2. To create a discrete material gradient scaffold, we merged the PCLA and PCLM together with chloroform. Scale bar = 500 μm.

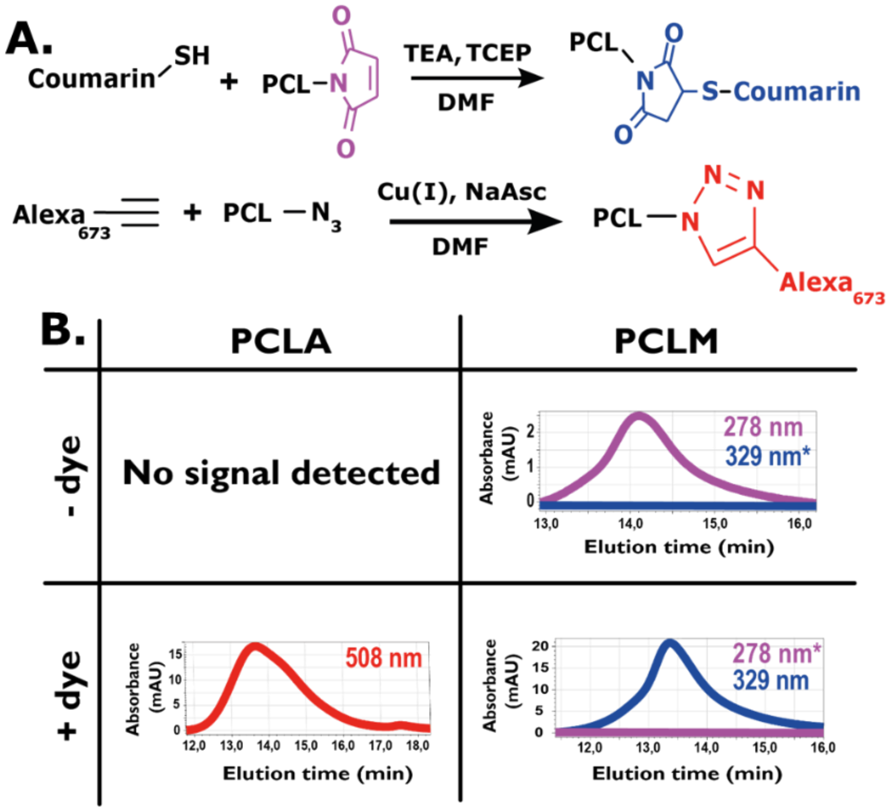

Figure S8. Reactions of PCLA and PCLM with fluorescent dyes in DMF. (A) Reaction schemes for a Michael addition with PCLM (top) and an alkyne-azide cycloaddition with PCLA (bottom). The colored group represents the targeted absorbing component that is detected by the photodiode array (PDA) detector. The selected wavelength (λ_{abs}) in the PDA detector for each of the corresponding groups is λ_{mal} = 279 nm, λ_{coum} = 329 nm, and $\lambda_{alexa673}$ = 508 nm. (B) Spectrophotometric analysis of emission wavelengths in SEC traces. SEC analysis confirmed successful coupling of the dye as the corresponding absorption was detected at the elution time of the polymer.

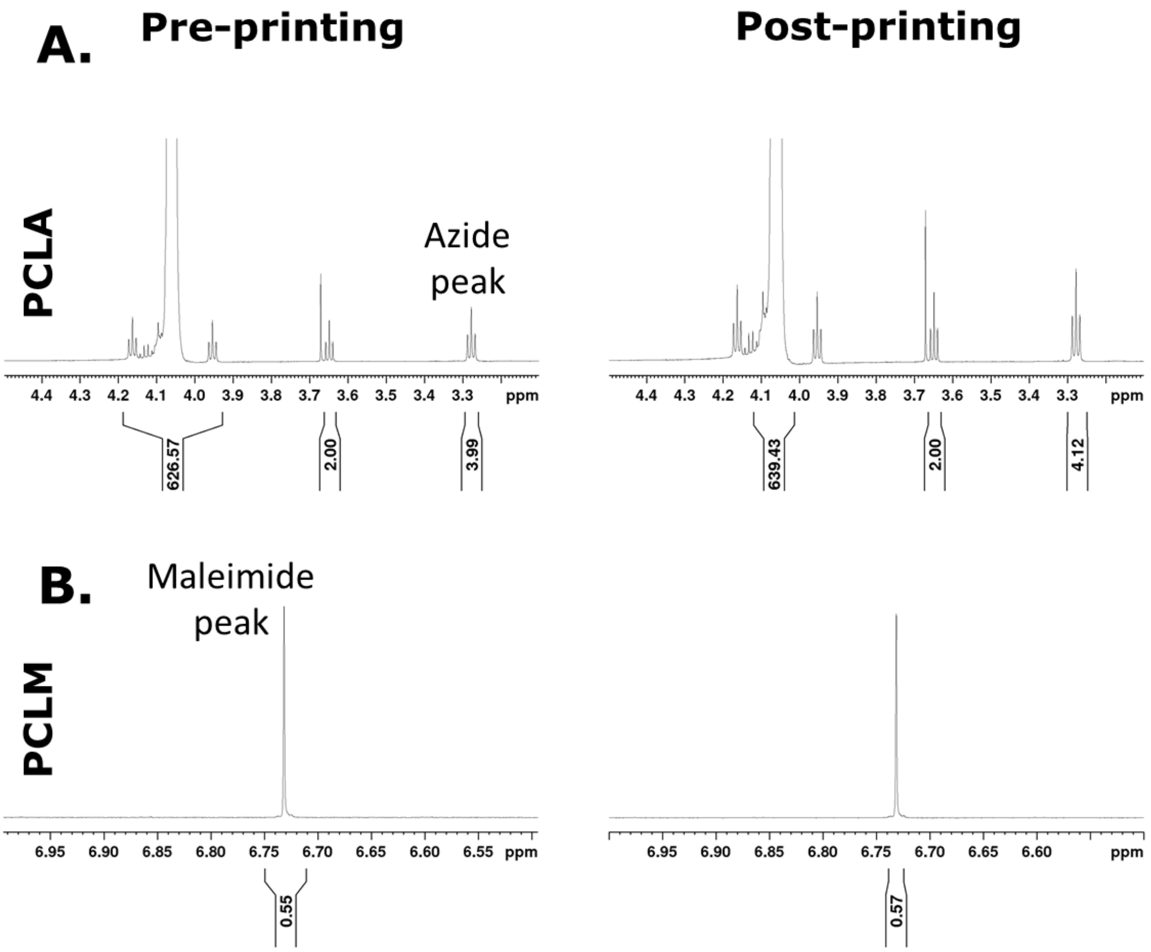

Figure S9. ^{1}H NMR spectra of PCLA and PCLM. A) Pre- and (B) post-printing ($CDCl_3$). No significant changes were observed in the integral values of the protons adjacent to the azide group or the vinylic protons of the maleimide group relative to the other signals.

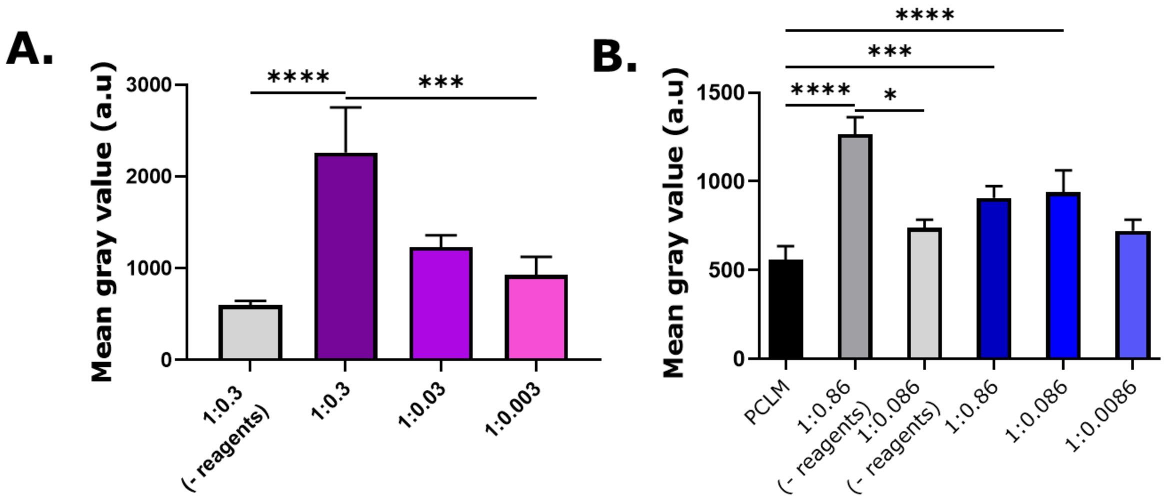

Figure S10. Fluorescence intensity at the surface of the scaffolds after click reactions and washing. We also included samples without reaction reagents. The mean gray value was determined at 5 different locations per scaffold. In (A) we displayed the intensity on the PCLA surface and in (B) the intensity on the PCLM. On the x-axis we placed the stoichiometric ratio between the total amount of azide/maleimide groups (in the bulk of the scaffold) and the added moles of dye, from high to low. Since groups are lost in the bulk, we overestimated the availability of the functional groups. The amount of azide/maleimide was set to 1. N=2 for samples, N=1 for the negative controls without reagents. *p<0.05, ***p<0.001, ****p<0.0001.

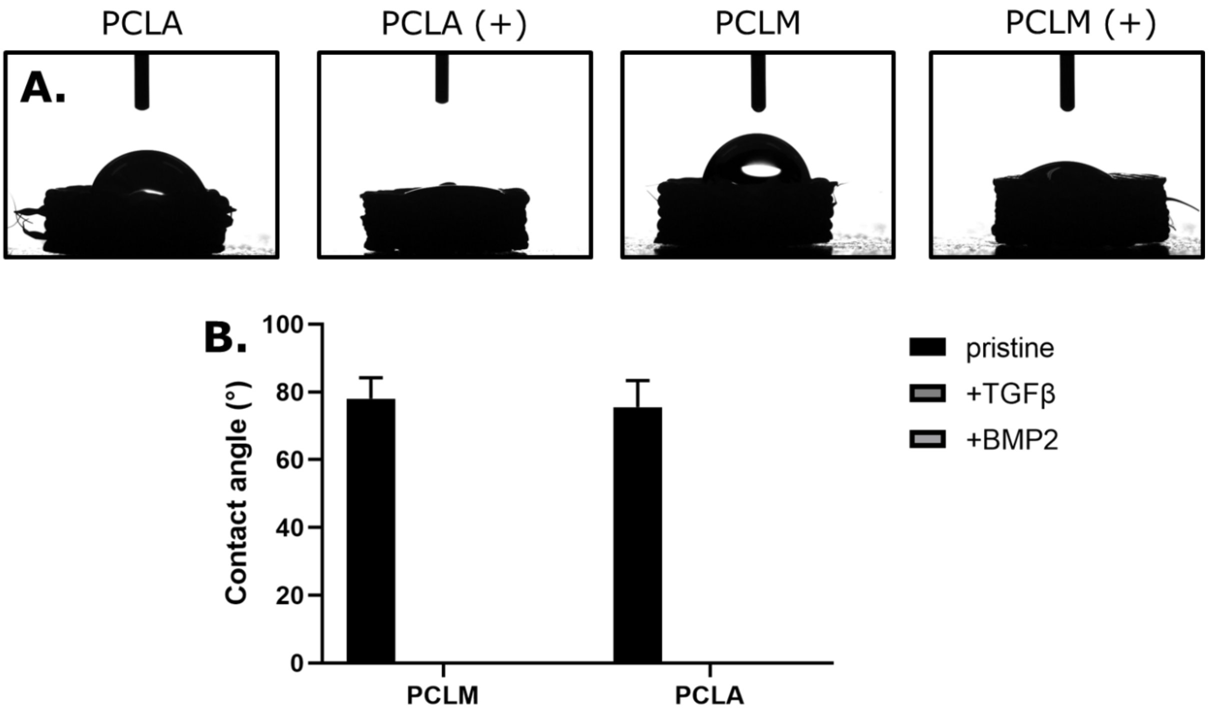

Figure S11. Water contact angle measurements using the sessile drop method (A) Images taken after 30 s. (B) Contact angles (N=3).

4. In-vitro hMSC study

4.1 Cell adhesion and proliferation

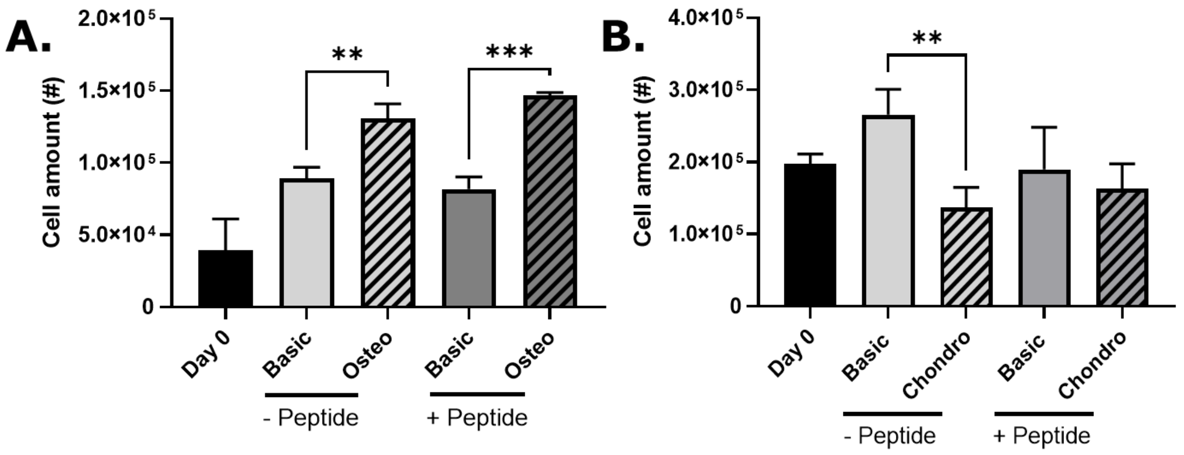

Figure S12. Cell numbers in osteogenic (A) and chondrogenic (B) constructs after 24 h (= Day 0) and 21 days. n=3. **p<0.01, ***p<0.001.

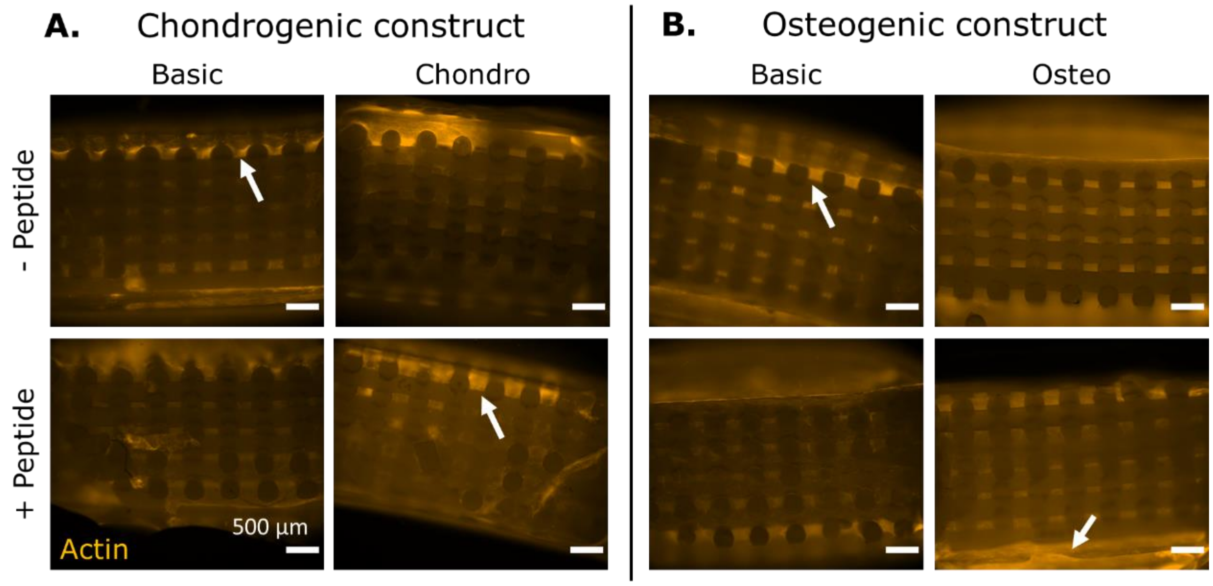

Figure S13. The cell distribution was determined by staining for actin (orange) in (A) chondrogenic and (B) osteogenic constructs in basic and differentiation medium. The arrows indicate areas where most cells were clustering.

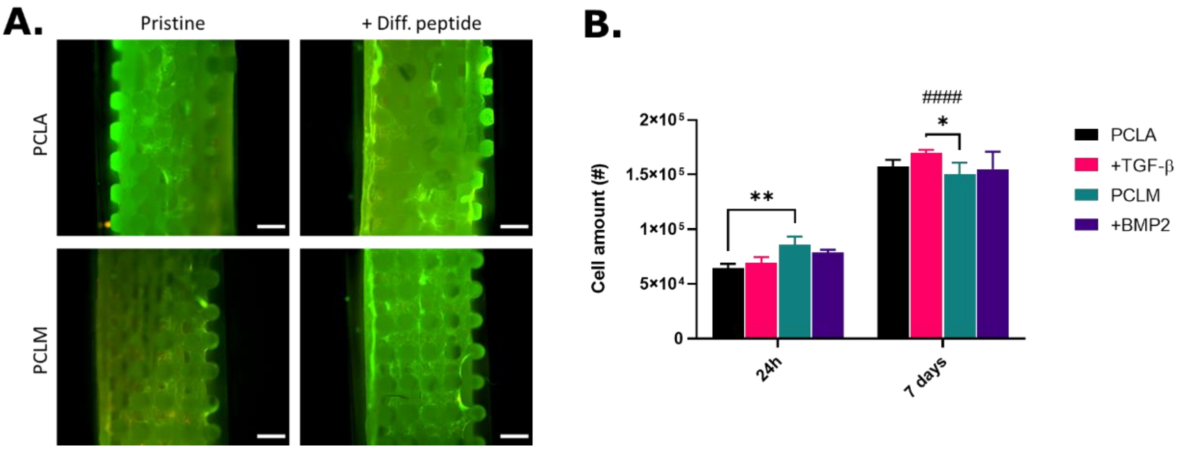

Figure S14. The cell distribution after 7 days (A) and cell numbers (B) in scaffolds seeded with dextran supplemented in the media after 24 h and 7 days. Scale bar = 500 µm. *$p<0.05$, **$p<0.01$. # was used to indicate significance in the cell amount when comparing 24 h vs 7 days. $^{\#\#\#\#}p<0.0001$.

4.2 Cell differentiation

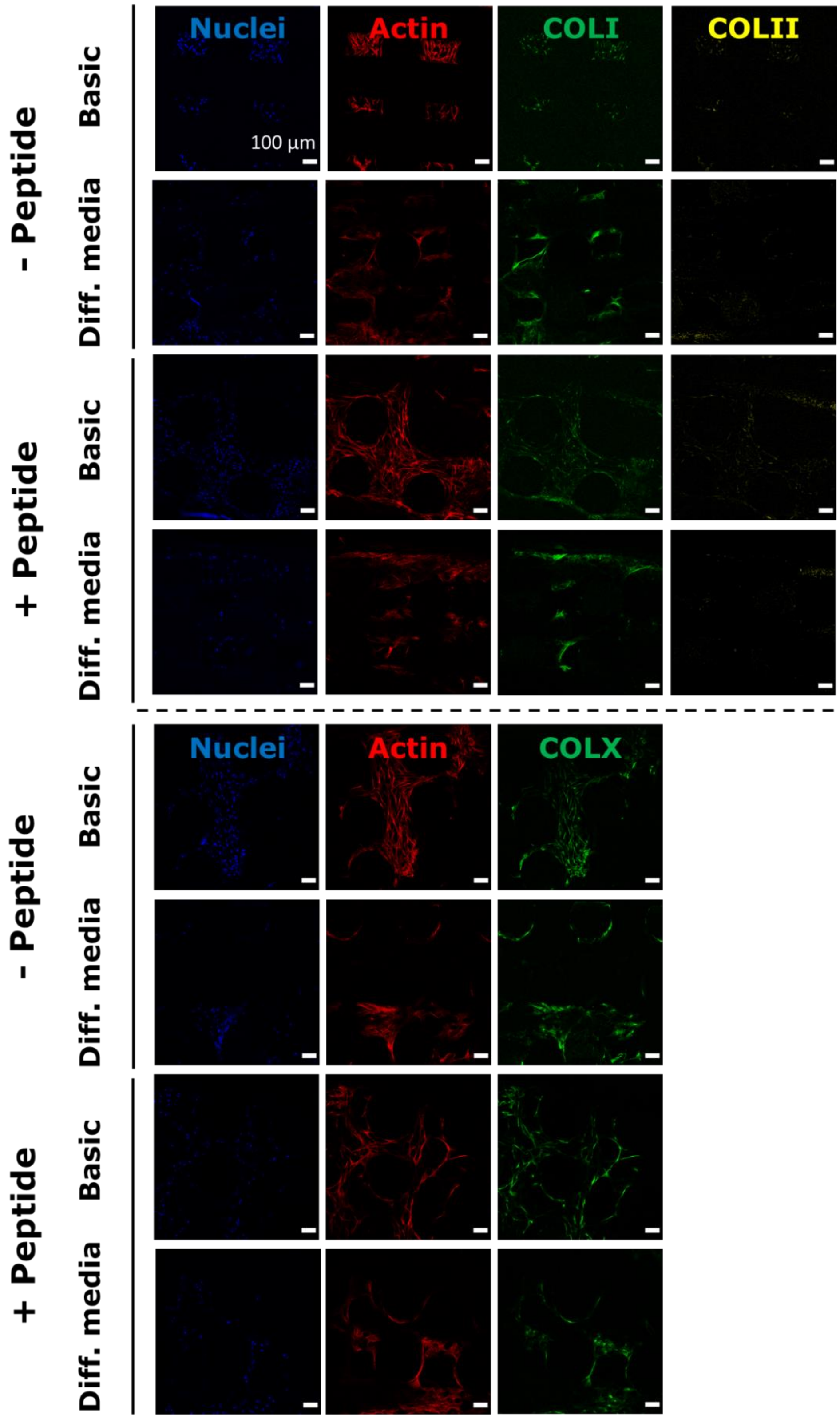

Figure S15. Split channel images of figure 3(A) displaying the collagen deposition of hMSCs after 21 days of culture in PCLA constructs. Nuclei were stained blue (DAPI) and actin red (phalloidin). In the top panel, we depicted the deposition of collagen I (green) and collagen II (yellow). In the bottom panel, we depicted the deposition of collagen X (green). Scale bar = 100 µm. N=2.

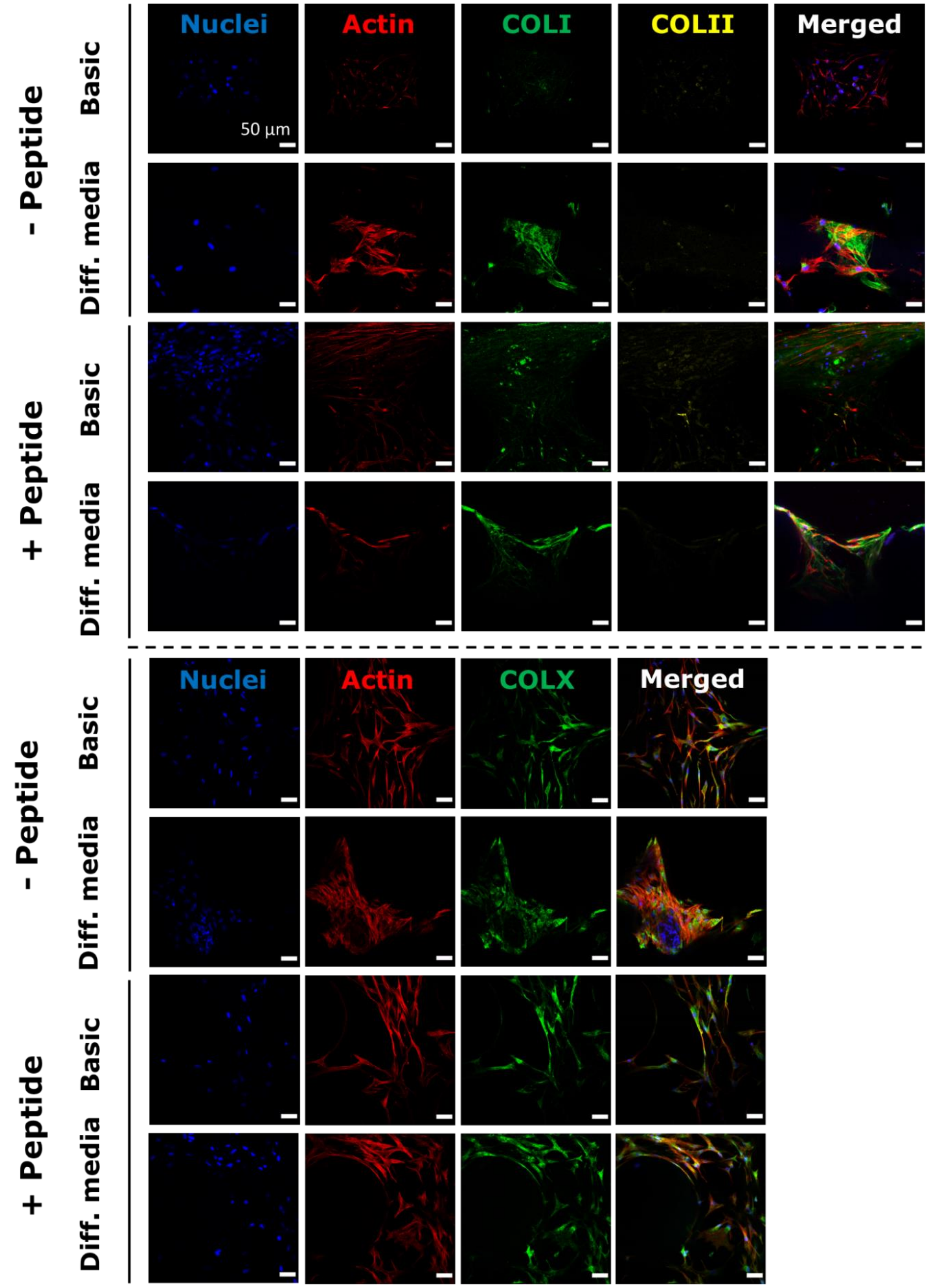

Figure S16. Higher magnification images of figure 3(A) displaying the collagen deposition of hMSCs after 21 days of differentiation in PCLA constructs. Nuclei were stained blue (DAPI) and actin red (phalloidin). In the top panel, we depicted the deposition of collagen I (green), collagen II (yellow), and merged images. In the bottom panel, we depicted collagen X (green) and merged images. Scale bar = 50 µm. N=2.

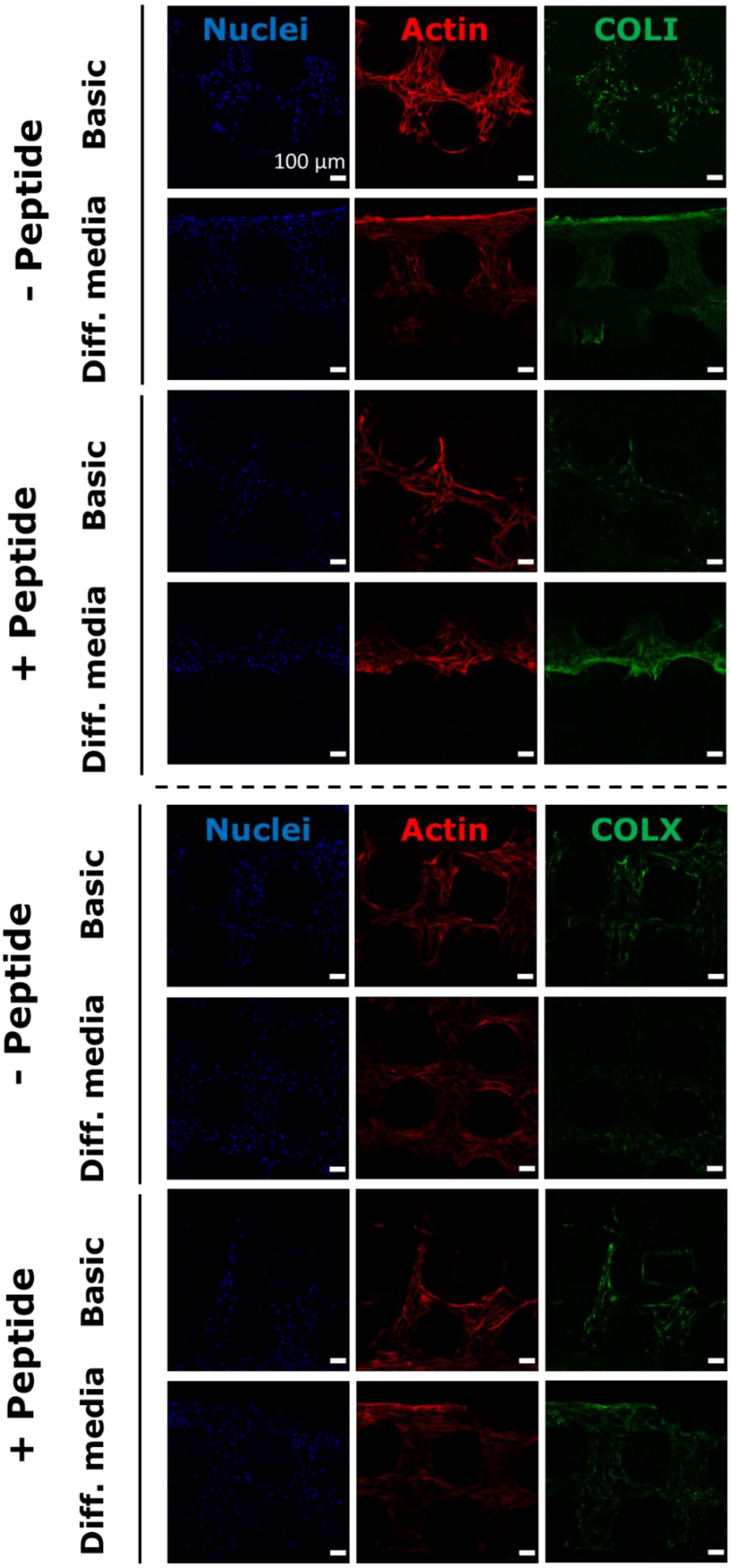

Figure S17. Split channel images of figure 3(B) displaying the collagen deposition of hMSCs after 21 days of culture in PCLM constructs. Nuclei were stained blue (DAPI) and actin red (phalloidin). In the top panel, we depicted the deposition of collagen I (green). In the bottom panel, we depicted the deposition of collagen X (green). Scale bar = 100 µm. N=2.

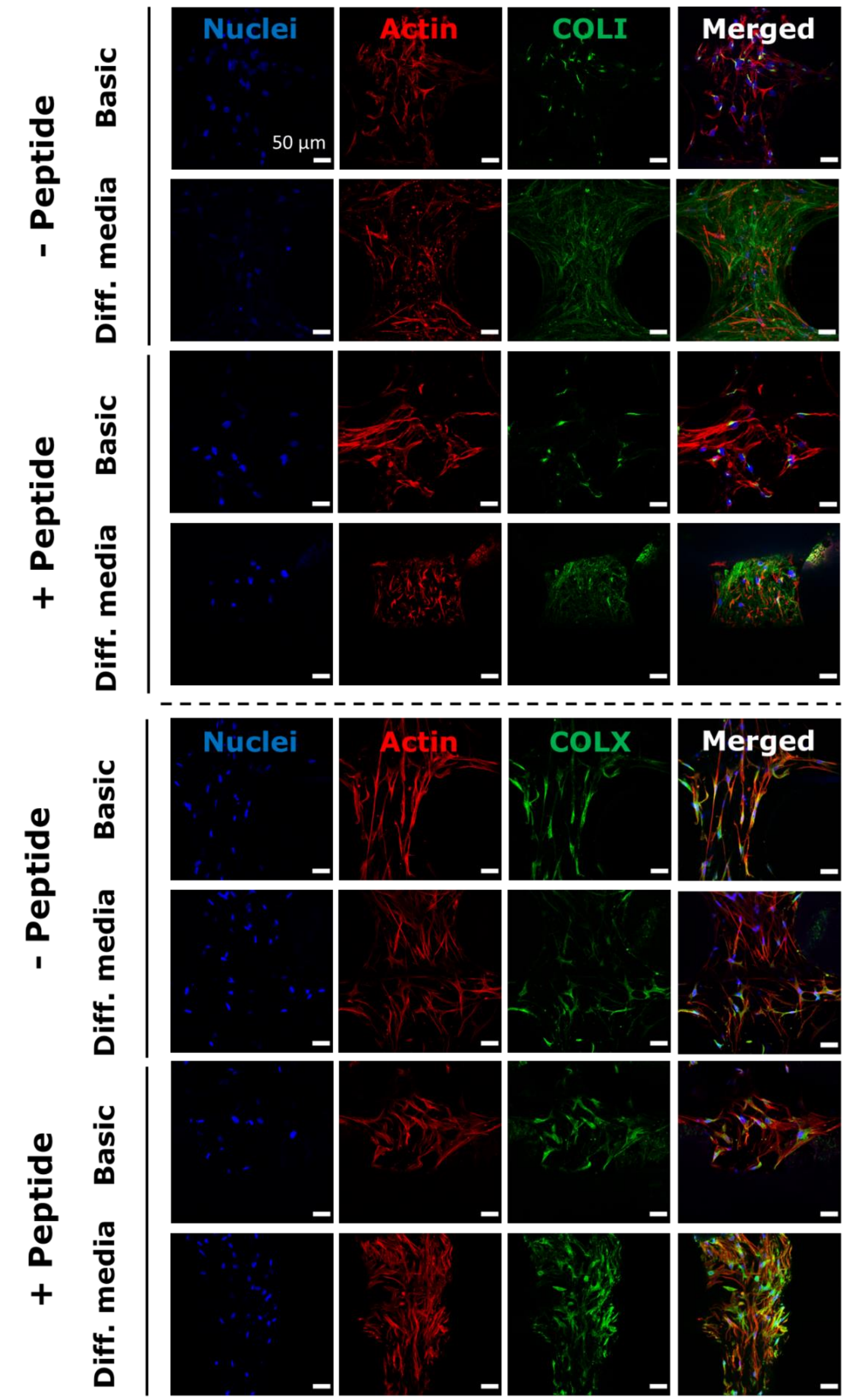

Figure S18. Higher magnification images of figure 3(B) displaying the collagen deposition of hMSCs after 21 days of differentiation in PCLM constructs. Nuclei were stained blue (DAPI) and actin red (phalloidin). In the top panel, we depicted the deposition of collagen I (green) and merged images. In the bottom panel, we depicted collagen X (green) and merged images. Scale bar = 50 μm. N=2.

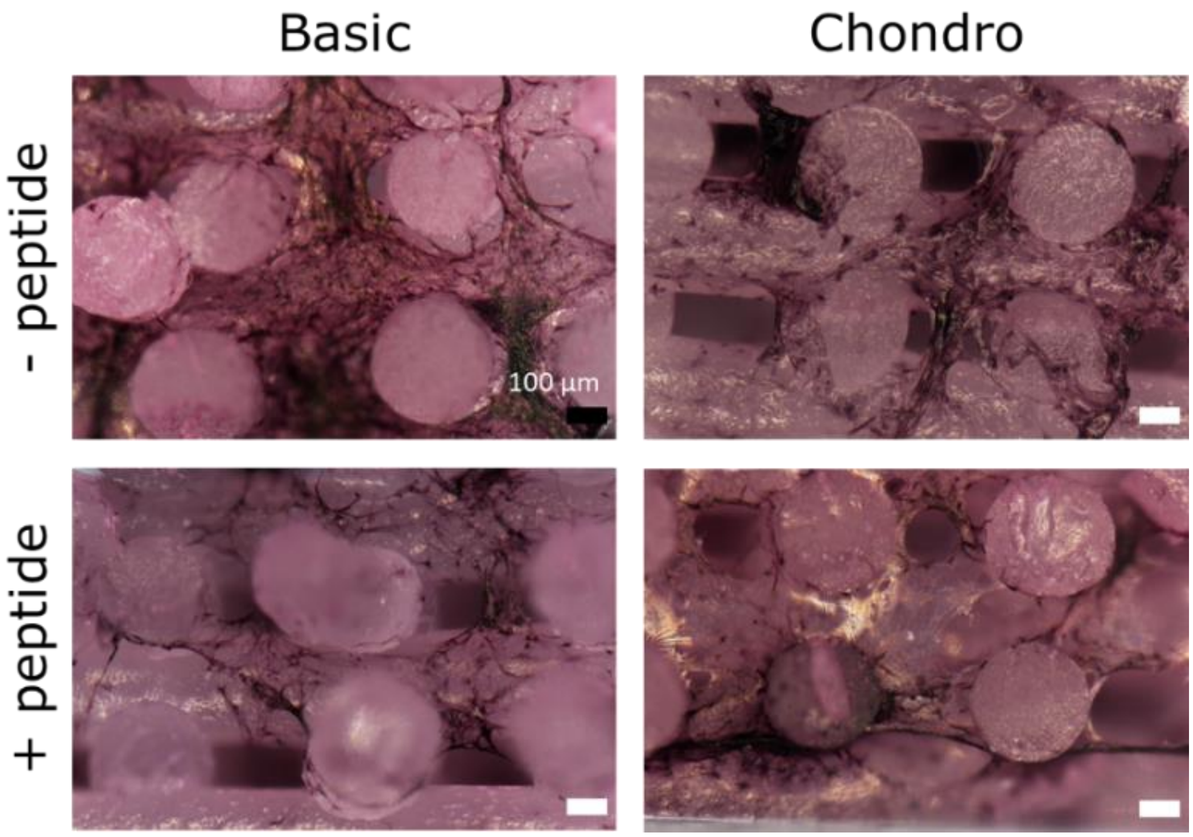

Figure S19. Safranin-O staining of cross sections of PCLA constructs with or without coupled chondro-peptide, after 21 days in basic and chondrogenic medium. Scale bar = 100 µm.

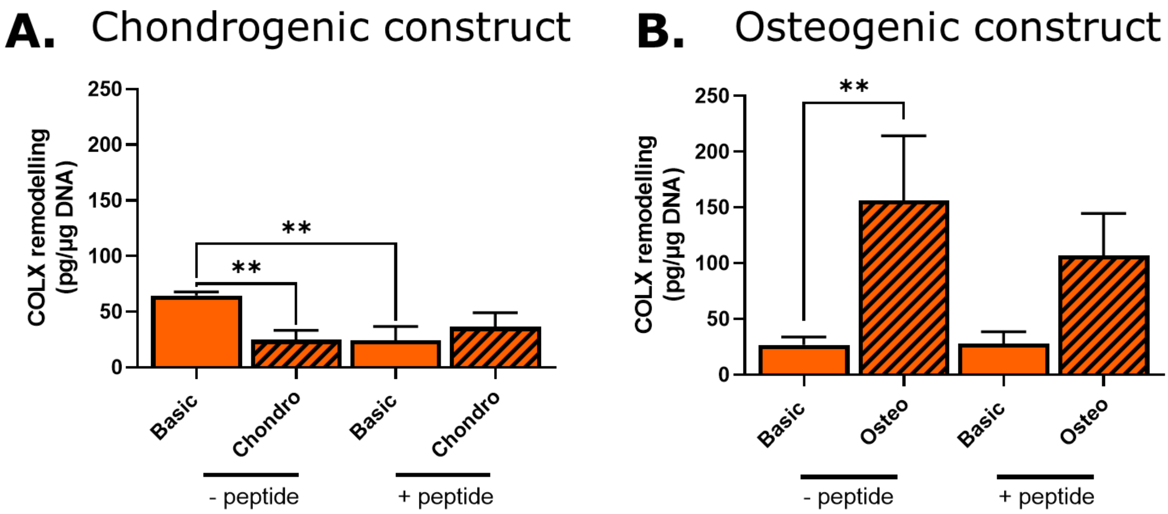

Figure S20. ELISA analysis of solubilized collagen X in the different media after 21 days in PCLA (A) or PCLM (B) constructs (with/without coupled peptide) as indication of collagen turnover. The amount of collagen X in the supernatant was normalized to the DNA content. N=3.

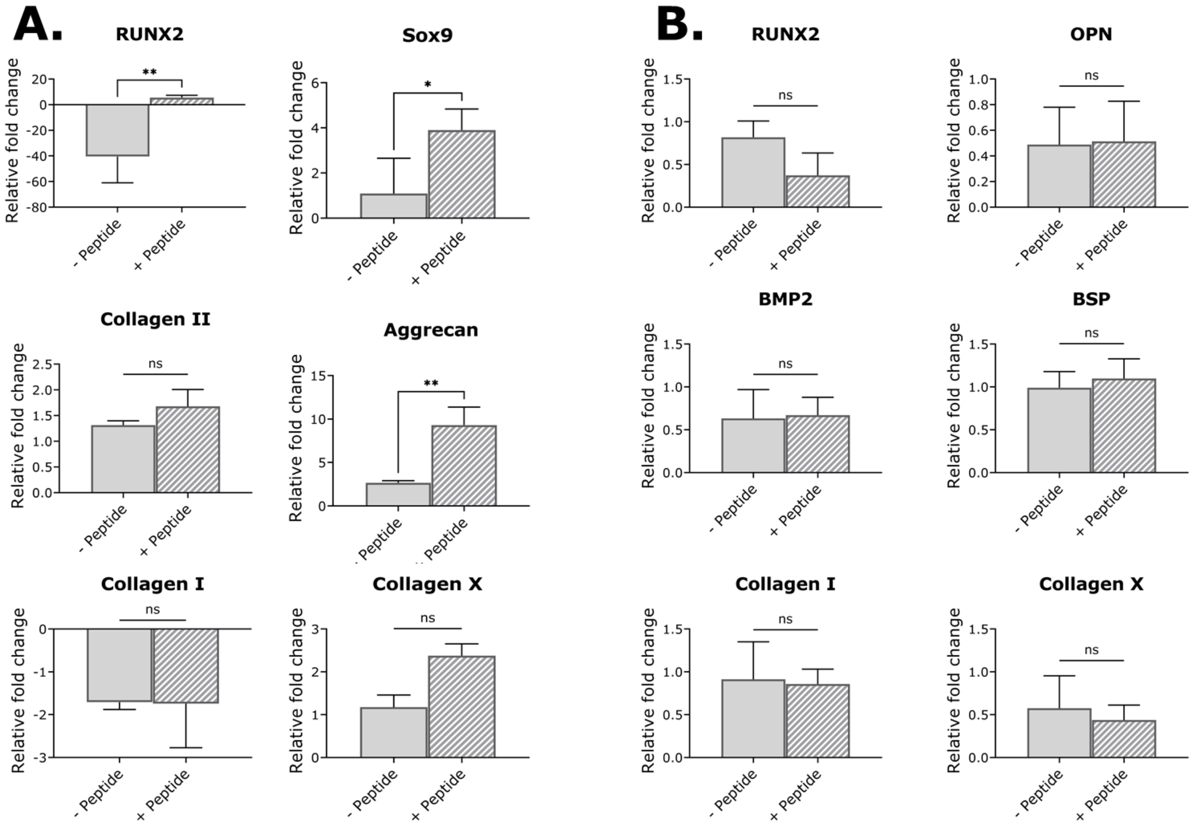

Figure S21. We determined the ratio of the fold change in scaffolds without peptide and with coupled peptide in BM to the corresponding fold change in differentiation media in figure 4. Panel (A) contains the chondrogenesis-related genes and panel (B) contains the osteogenesis related genes. N => 2. *p<0.05, **p<0.01.

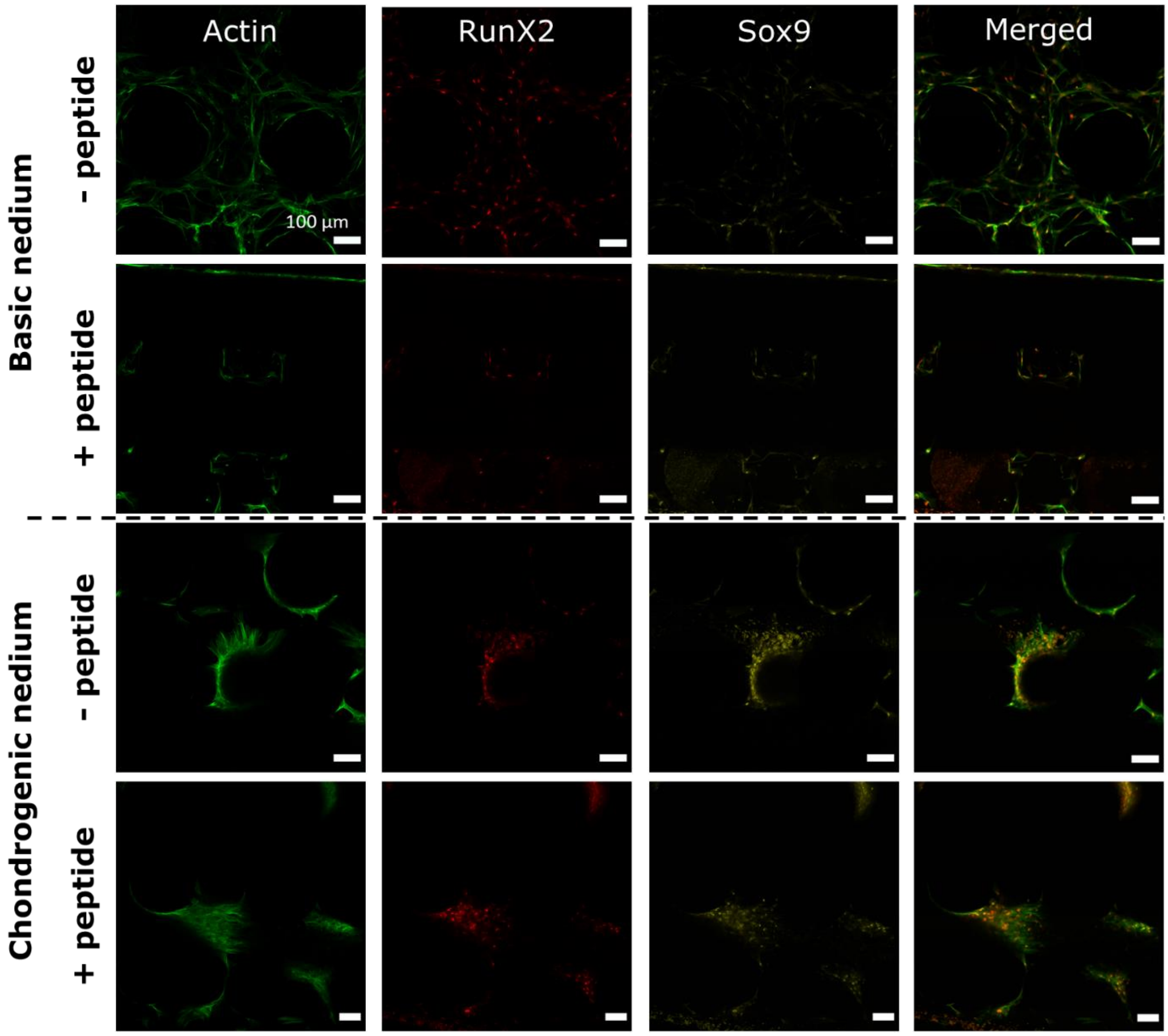

Figure S22. Immunofluorescent staining of actin (green), RunX2 (red), and Sox9 (yellow) after 7 days in chondrogenic constructs. Constructs were incubated in basic medium (top panel) and chondrogenic medium (bottom panel). Scale bar = 100 µm. N=2.

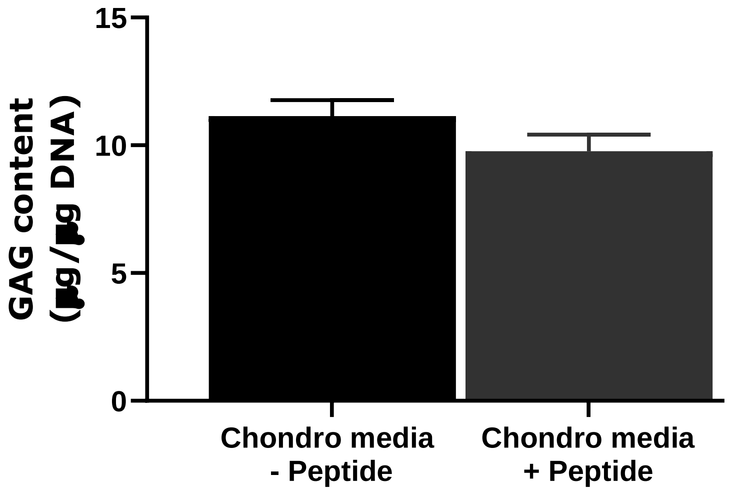

Figure S23. GAG content normalized to DNA content in chondrogenic medium after 21 days. In this experiment, cells were seeded in dextran supplemented medium to enhance cell distribution. N=3.

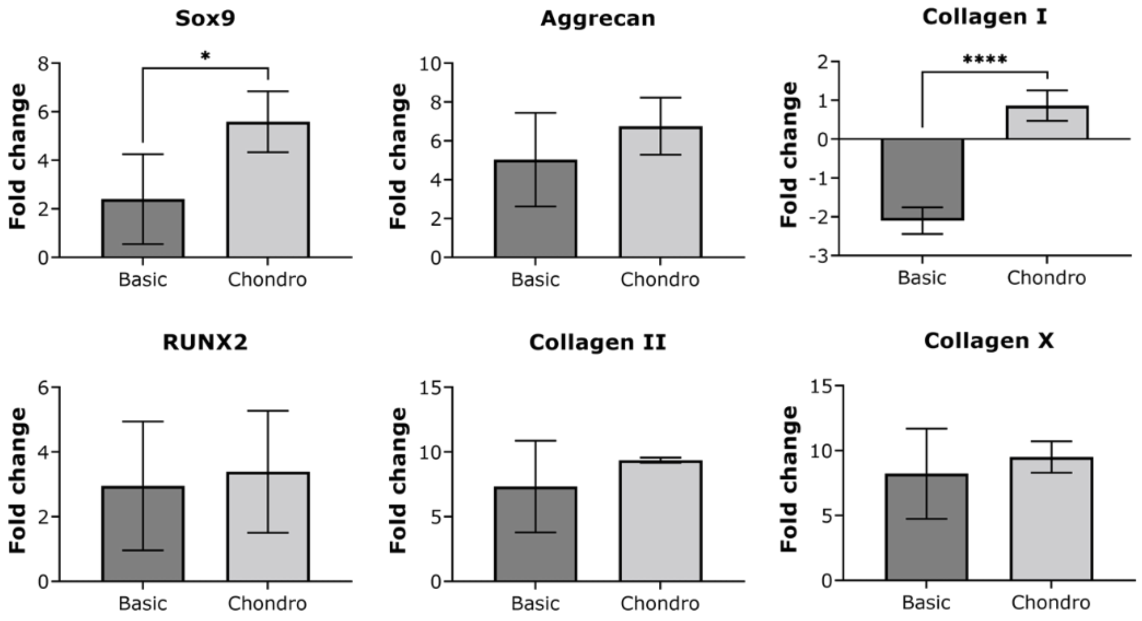

Figure S24. Fold change in relative gene expression levels in the chondro pellets after 21 days. The fold change was determined relatively to cells in monolayer culture. N => 2. *p<0.05, ****p<0.0001.

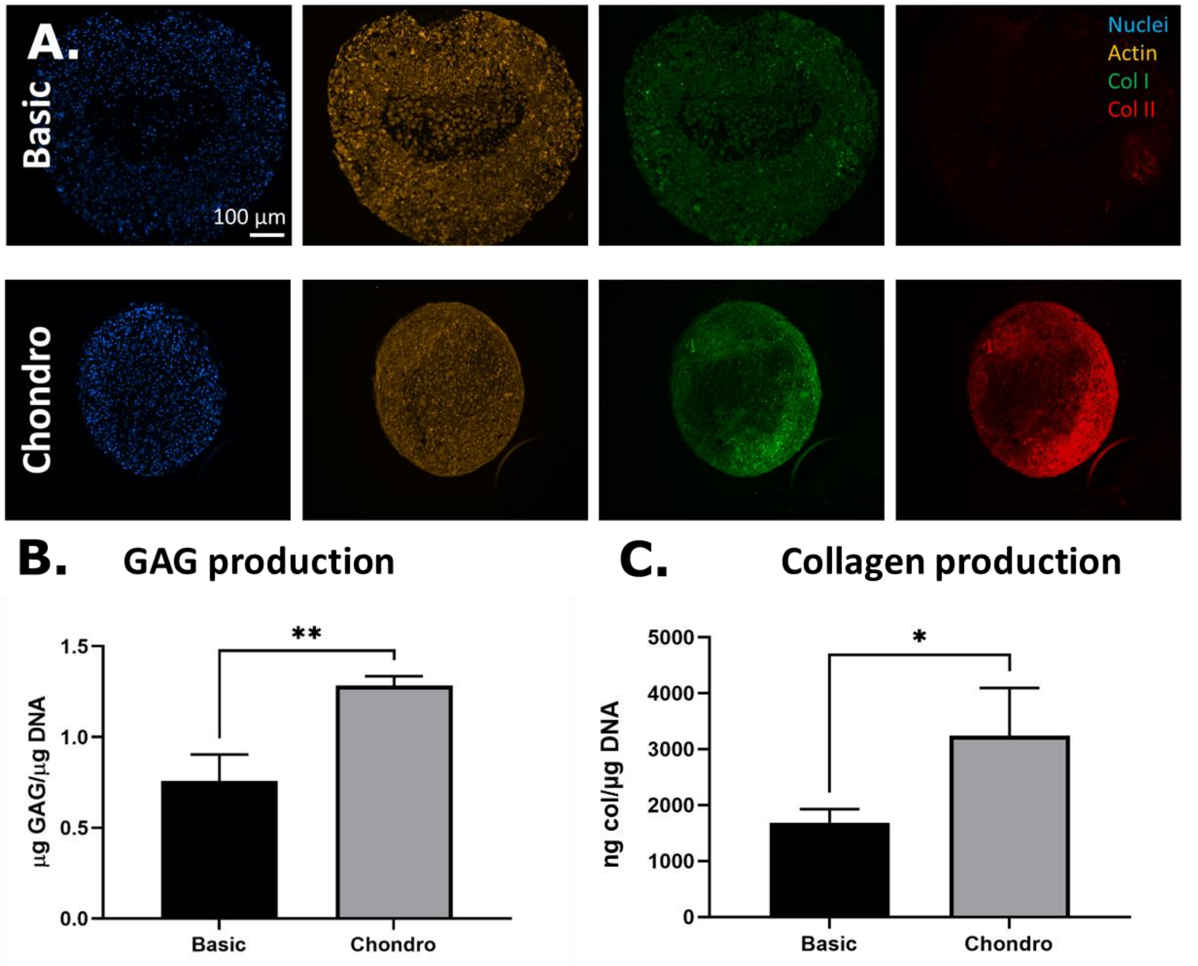

Figure S25. ECM deposition of the chondro pellets after 21 days in basic or chondrogenic medium. Panel (A) depicts cross sections of the pellets in basic and chondrogenic media, stained for nuclei (blue), actin (yellow), collagen I (green), and collagen II (red). Scale bar = 100 µm. N=2. The normalized GAG (B) and collagen (C) content (N = 3). $^*p<0.05$, $^{**}p<0.01$.

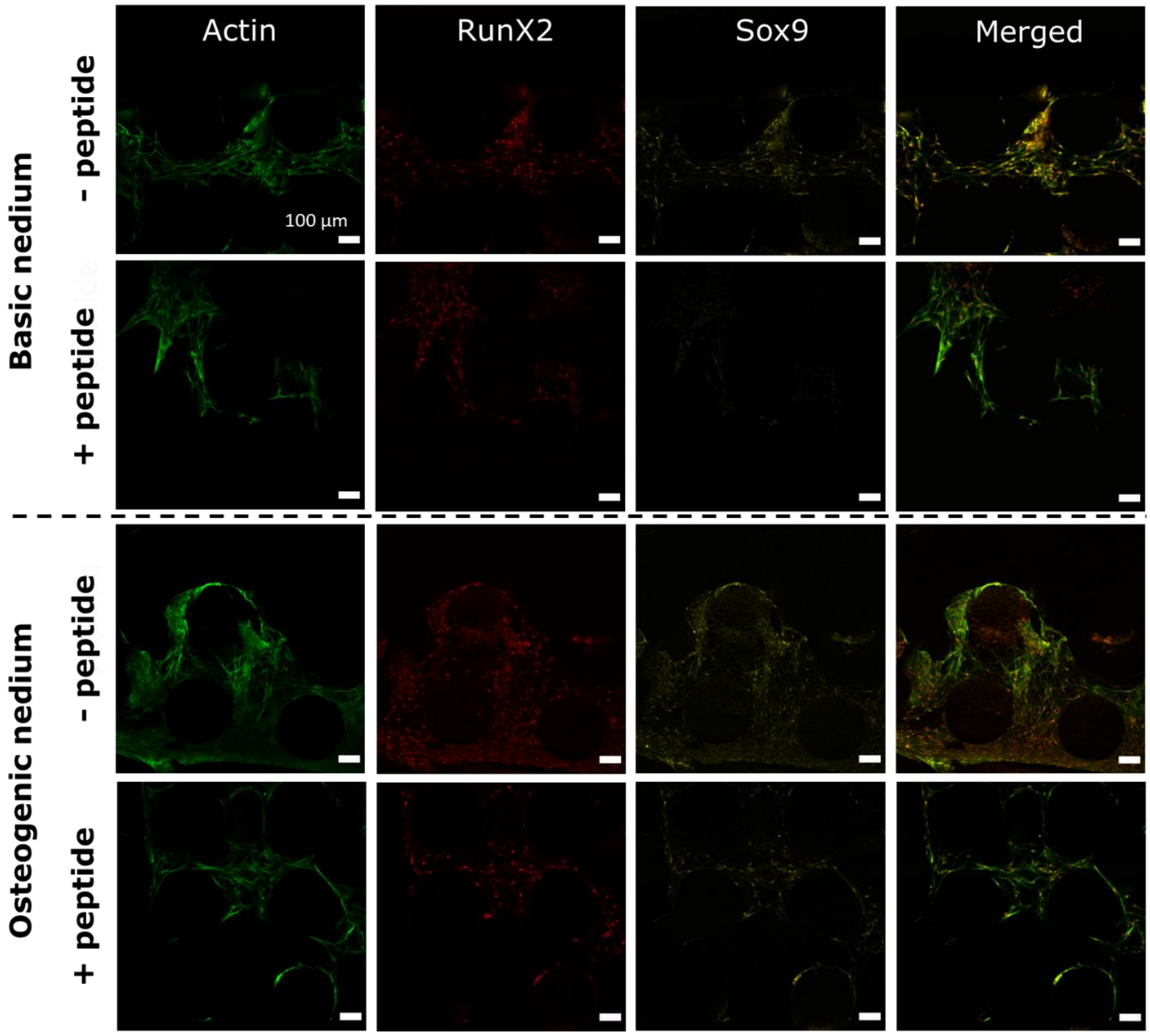

Figure S26. Immunofluorescent staining of actin (green), RunX2 (red), and Sox9 (yellow) after 7 days in osteogenic constructs. In the top panel, constructs were incubated in basic medium and, in the bottom panel, constructs were incubated in chondrogenic medium. Scale bar = 100 µm. N=2.

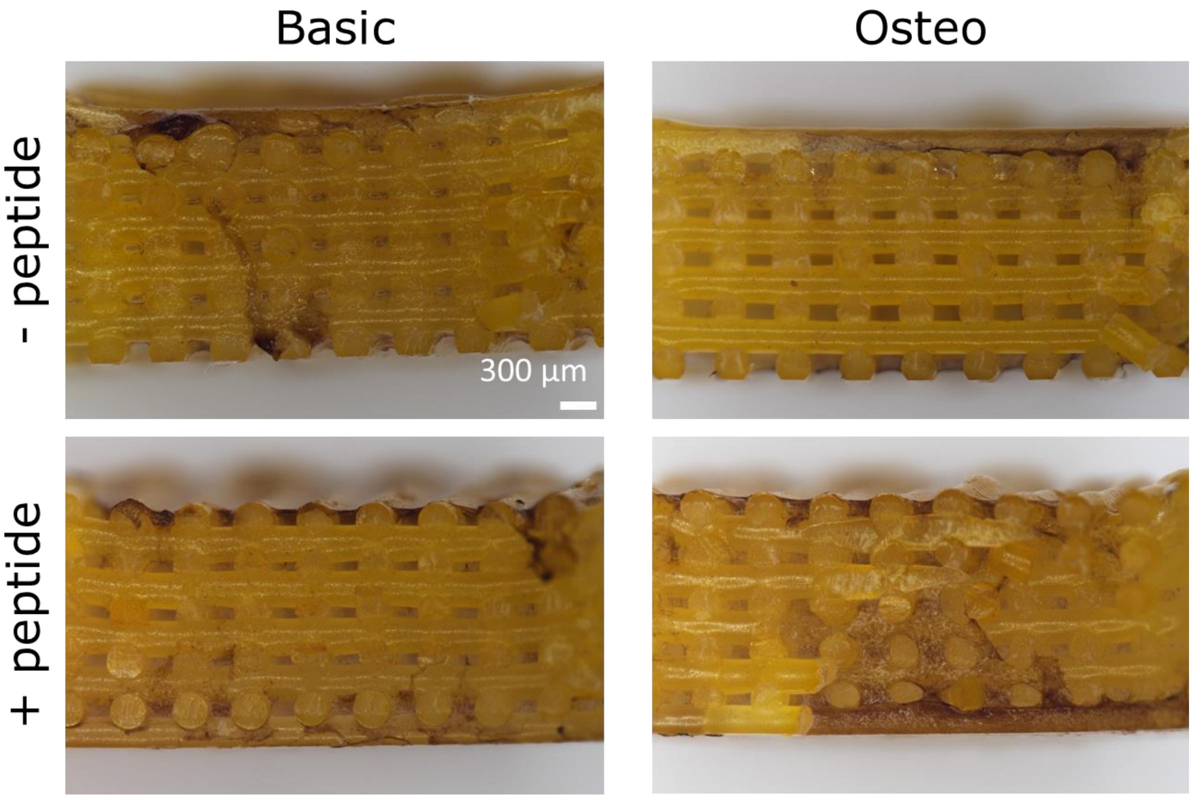

Figure S27. Mineralization pockets in the osteogenic constructs after 21 days of culture. Scale bar = 300 µm. N = 2.

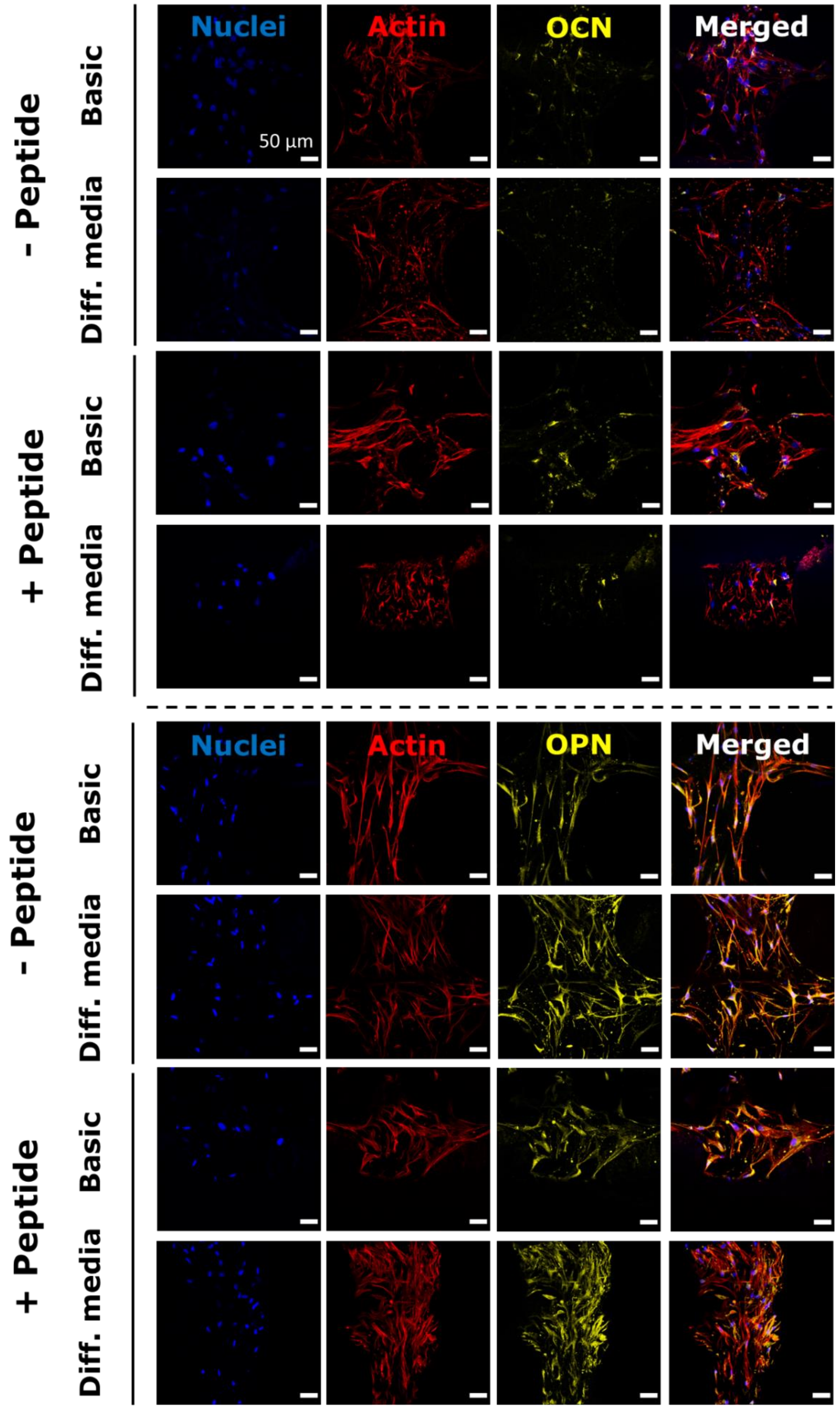

Figure S28. Images of cross sections of osteogenic constructs stained for osteocalcin (top panel) and osteopontin (bottom panel) in yellow. All constructs were stained for the nuclei (blue) and actin (red). Scale bar = 50 μm. N=2.

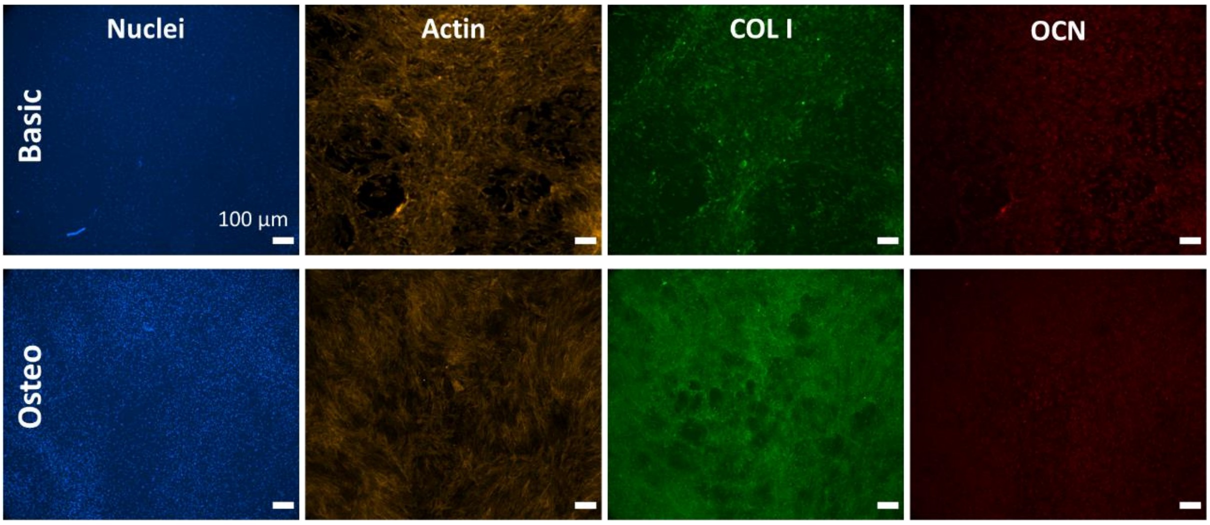

Figure S29. 2D cultured cells in basic and osteogenic medium, stained for nuclei (blue), actin (yellow), collagen I (green), and osteocalcin (red) after 21 days. Scale bar = 100 µm. N = 2.

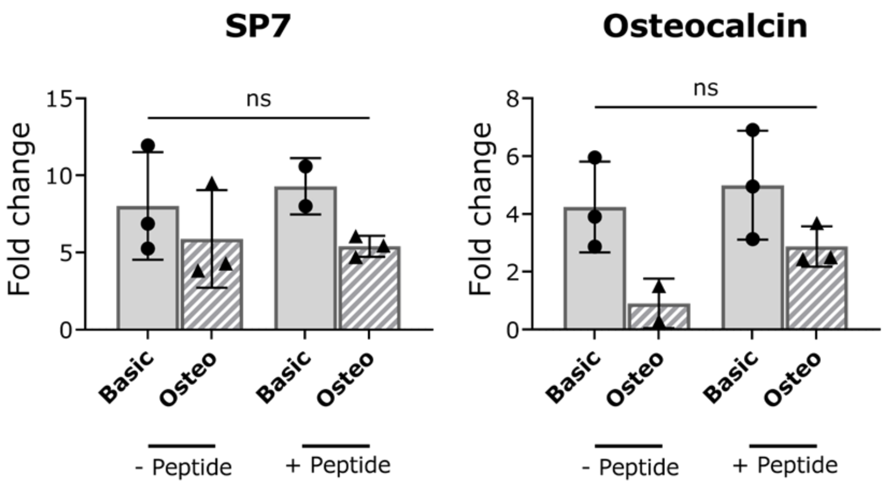

Figure S30. Fold change in relative gene expression levels of (left) osteocalcin and (right) Sp7 in the osteogenic constructs after 21 days. We determined the fold change relatively to the cells in monolayer culture before the experiment. N = 2. Ns = non-significant.

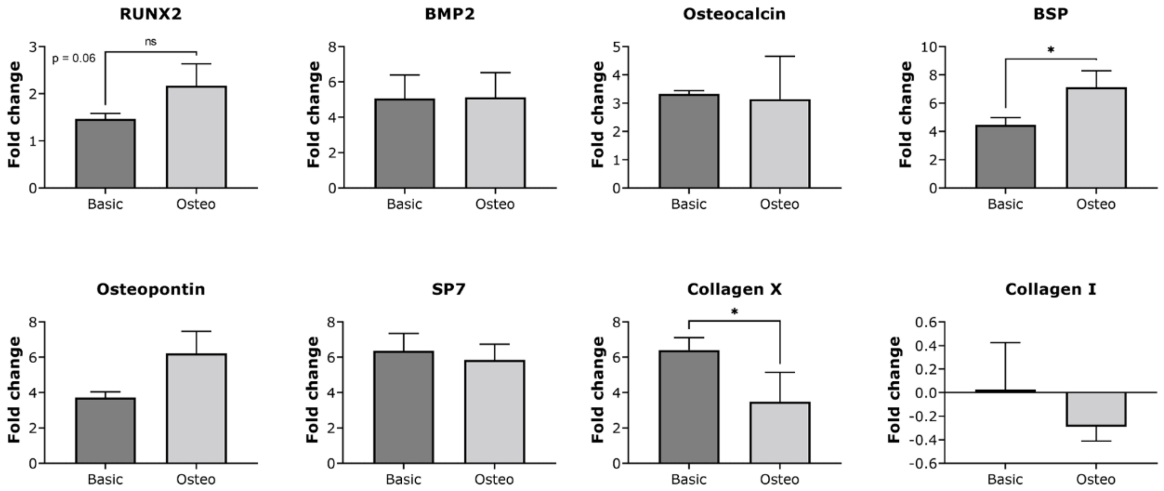

Figure S31. Fold change in relative gene expression levels of osteogenesis-related genes in the 2D cultured hMSCs after 21 days. We determined the fold change relatively to the cells in monolayer culture before the experiment. N => 2. *p<0.05.

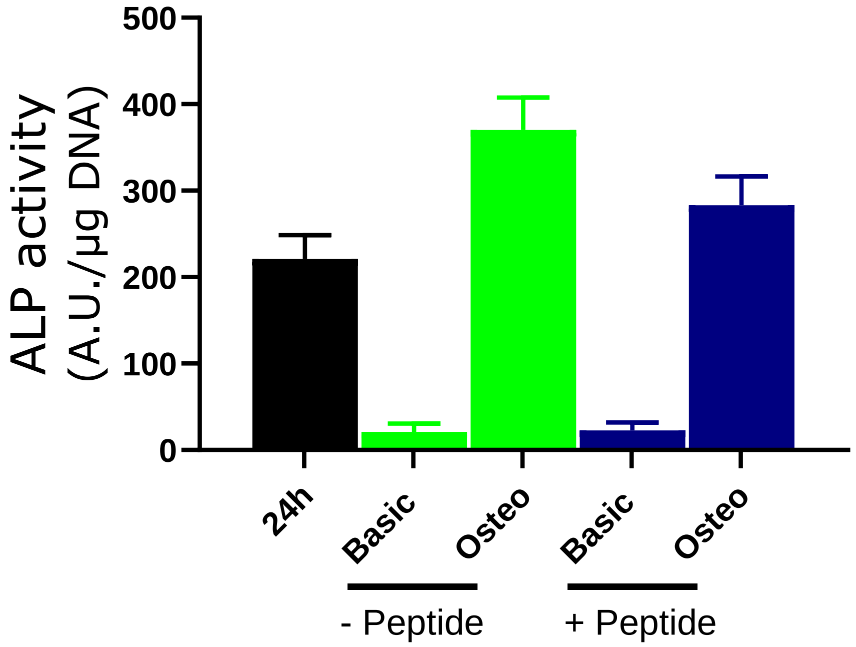

Figure S32. ALP activity normalized to DNA content in the chondrogenic medium after day 21. In this experiment, cells were seeded in dextran supplemented medium to enhance cell distribution. N=3.

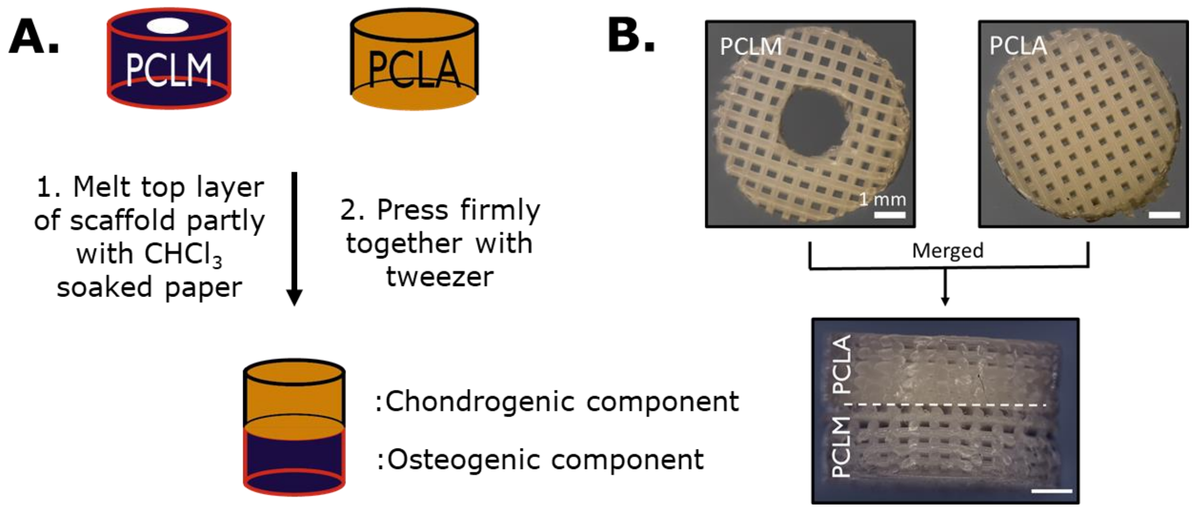

Figure S33. Creating a discrete material (biphasic) construct. (A) Protocol to create biphasic construct, which contains a chondrogenic (PCLA +/- chondro-peptide) and osteogenic (PCLM +/- osteo-peptide) component. (B) Stereomicroscope images of the merged components.

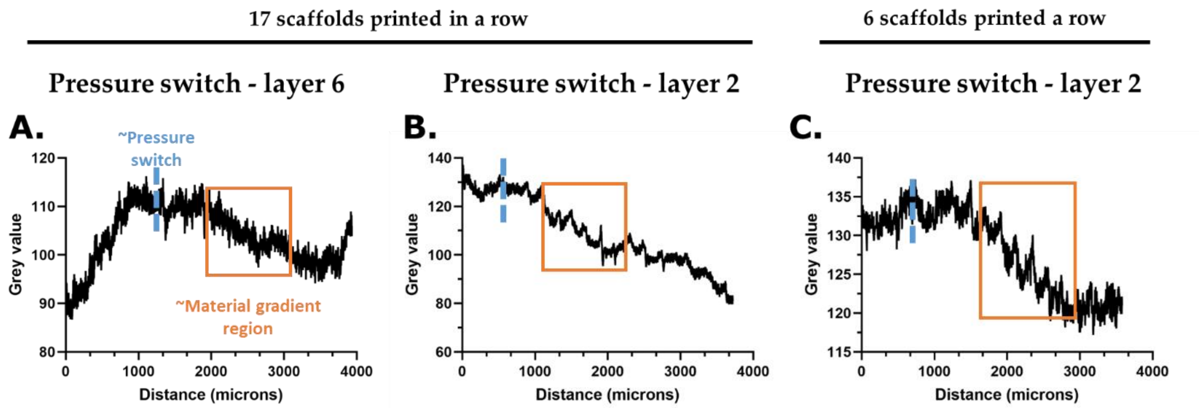

Figure S34. Gray intensity profiles as a function of scaffold height, from top to bottom (PCLA to PCLM). The blue dashed line represents the transition of the pressure from one reservoir to the other. After a short delay, the dye starts to appear. The orange box displays the location of the gradient. After the orange box a plateau is reached.

Assumptions:

- Rounded fibers
- Fiber height (as set by g-code) = layer height = 200 µm

$V_{1\ layer}$ = ~0.0028 cm^3

Gradient length = Distance from pressure switch signal until plateau (µm)

The volume to estimate the volume to switch materials follows from:

$$V_{\text{transition}} = \frac{\text{Layers}}{\text{Fiber height}} \times \#_{printed\ scaffolds} \times V_{1layer}$$

Figure S35. Assumption and calculations to determine the volume to switch from one material to another with the duo-reservoir print head.

5. Tables

Table S1. List of primers used for RT-PCR experiments

Gene	Forward primer 5'to 3'	Reverse primer 5'to 3'
GAPDH	ATG GGG AAG GTG AAG GTC G	TAA AAG CAG CCC TGG TGA CC
Coll2a1	GGCAATAGCAGGTTCACGTACA	CGATAACAGTCTTGCCCCACTT
Coll1a1	AGGGCCAAGACGAAGACATC	AGATCACGTCATCGCACAACA
Sox9	TTC CGC GAC GTG GAC AT	TCA AAC TCG TTG ACA TCG AAG GT
ColXa1	GAC TCC CTC CTC ACT GTC GC	AGG GAA GTC TCC CTC ACT TGT
ACAN	GCCTTGAGCAGTTCACCTTC	CTCTTCTACGGGGACAGCAG
RunX2	AGT GAT TTA GGG CGC ATT CCT	GGA GGG CCG TGG GTT CT
BSP	CCCCACCTTTTGGGAAAACCA	TCCCCGTTCTCACTTTCATAGAT
OCN	TGAGAGCCCTCACACTCCTC	CGCCTGGGTCTCTTCACTAC
SP7	CCTCTGCGGGACTCAACAAC	AGCCCATTAGTGCTTGTAAAGG
BMP2	ACTACCAGAAACGAGTGGGAA	GCATCTGTTCTCGGAAAACCT
OPN	CTCCATTGACTCGAACGACTC	CAGGTCTGCGAAACTTCTTAGAT

Table S2. Architectural and mechanical analysis of the scaffolds.

Condition	Fiber diameter (μm)		Pore size (μm^2)	Layer height (μm)
PCLA	289 ± 5.5		$66.6 \cdot 10^3 \pm 5.5 \cdot 10^3$	279 ± 3.2
PCLM	280 ± 7.1		$68.4 \cdot 10^3 \pm 4.7 \cdot 10^3$	254 ± 4.7
Discrete gradient[a]				
Continuous gradient	272 ± 9.6[b]	280 ± 13[c]	$71.4 \cdot 10^3 \pm 7.9 \cdot 10^3$	274 ± 19

[a]Discrete gradient scaffolds were PCLA and PCLM merged by chloroform. [b]PCLA and [c]PCLM side of the continuous gradient construct.

Table S3. Dye surface density on PCLA fibers of scaffolds determined by spectrofluorimetric measurements. The concentration of dye present in 30 mg of scaffold was determined from a standard curve. Based on the scaffold mass and estimated surface area, the surface density was calculated in this representative spreadsheet.

Stoichiometry[a]	Intensity (A.U.)[b]	Dye Concentration (nM)[c]	Amount of moles (nmol)[d]	Total nmol in the full scaffold[e]	Surface density (nmol/cm²)[f]	Surface density (pmol/cm²)
1:1	321	136.869	0.137	0.202	0.036	36.35
10:1	95.2	37.310	0.037	0.055	0.010	9.86
100:1	92.3	36.032	0.036	0.051	0.009	9.16
1:1 (- reagents)	33.2	9.974	0.010	0.014	0.002	2.48
PCLA only	3.40	3.754	0.004	0.005	0.001	0.90

[a]Dye:Azide molar ratio (according to the amount of azides present in the bulk of the scaffold).
[b]Values of 30 mg PCLA scaffold in 1 mL $CHCl_3$ (after reaction) were recorded at λ_{em} = 642 nm via the spectrofluorometer.
[c]Calculated from a standard curve; this is the concentration of dye in the measured sample.
[d]Scaffolds were dissolved at 30 mg/mL for this analysis. A volume of 1 mL was used.

$n_{dye} = C_{sample}*V_{analysis}$; where n_{dye} is the amount of nmol dye bound in the measured sample; we assumed that all the measured dye was bound to the surface, C_{sample} is the concentration determined from a standard curve, and $V_{analysis}$ is the volume used for analysis.

[e]The average total scaffold of PCLA scaffolds mass was 40 mg. As we only used 30 mg of the full scaffold, we corrected the nmol of dye to estimate the amount on the surface of one full scaffold.

$n_{tot.scaffold} = n_{dye}/(m_{analysis}/m_{scaffold})$, where $m_{analysis}$ = 30 mg, $m_{scaffold}$ = 40 mg, n_{30mg} = nmol of dye on 30 mg.

fThe surface area of the full scaffold was estimated via $\pi*d_{fiber}*l_{fiber}$; where d_{fiber} was measured and rounded to 300.10^{-2} cm, l_{fiber} is the length of the fiber in the full scaffold. From the g-code we extracted l_{fiber} = 59 cm. Thus, the surface area ($A_{surface}$) equals 5.56 cm^2. In the surface density calculation, we assumed that no area was lost due to fiber-fiber contact.

Surface density = $n_{tot.scaffold}/A_{surface}$ ([nmol]/[cm^2]).

Table S4. Dye surface density on PCLM fibers of scaffolds determined by spectrofluorimetric measurements. The concentration of dye present in 30 mg of scaffold was determined from a standard curve. Based on the scaffold mass and estimated surface area, the surface density was calculated in this representative spreadsheet.

Stoichiometry[a]	Intensity (A.U.)[b]	Dye Concentration (nM)[c]	Amount of moles (nmol)[d]	Total nmol in the full scaffold[e]	Surface density (nmol/cm²)[f]	Surface density (pmol/cm²)
1:1	80.40	92.16	0.092	0.113	0.020	20.39
10:1	26.70	38.06	0.038	0.049	0.009	8.89
100:1	45.00	56.49	0.056	0.072	0.013	13.04
1:1 (- reagents)	66.30	77.95	0.078	0.097	0.018	17.53
10:1 (-reagents)	36.40	47.83	0.048	0.060	0.011	10.88
PCLM only	4.54	15.73	0.016	0.020	0.004	3.54

[a]Dye:Maleimide molar ratio (according to the amount of maleimides present in the bulk of the scaffold).
[b]Values of 30 mg PCLM scaffold in 1 mL $CHCl_3$ (after reaction) were recorded at λ_{em} = 525 nm via the spectrofluorometer.
[c]Calculated from a standard curve; this is the concentration of dye in the measured sample.

[d]Scaffolds were dissolved at 30 mg/mL for this analysis. A volume of 1 mL was used.

$n_{dye} = C_{sample}*V_{analysis}$; where n_{dye} is the amount of nmol dye bound in the measured sample; we assumed that all the measured dye was bound to the surface, C_{sample} is the concentration determined from a standard curve, and $V_{analysis}$ is the volume used for analysis.

[e]The average total scaffold of PCLM scaffolds mass was 37 mg. As we only used 30 mg of the full scaffold, we corrected the nmol of dye to estimate the amount on the surface of one full scaffold.

$n_{tot.scaffold} = n_{dye}/(m_{analysis}/m_{scaffold})$, where $m_{analysis}$ = 30 mg, $m_{scaffold}$ = 37 mg, n_{30mg} = nmol of dye on 30 mg.

fThe surface area of the full scaffold was estimated via $\pi*d_{fiber}*l_{fiber}$; where d_{fiber} was measured and rounded to 300.10^{-2} cm, l_{fiber} is the length of the fiber in the full scaffold. From the g-code we extracted l_{fiber} = 59 cm. Thus, the surface area ($A_{surface}$) equals 5.56 cm^2. In the surface density calculation, we assumed that no area was lost due to fiber-fiber contact.

Surface density = $n_{tot.scaffold}/A_{surface}$ ([nmol]/[cm^2]).
